# Design and Validation of a Multi-Epitope mRNA Vaccine Construct Against Human Monkeypox Virus (hMPXV) by Annotating Protein of Intracellular Mature Virus (IMV) Form of hMPXV

**DOI:** 10.3390/biomedicines13061439

**Published:** 2025-06-11

**Authors:** Mohammad Asrar Izhari, Siraj B. Alharthi, Raed A. Alharbi, Ahmad H. A. Almontasheri, Wael A. Alghamdi, Abdulmajeed Abdulghani A. Sindi, Ahmad Abdulmajed Salem, Ali Mahzari, Fahad Alghamdi, Ahmed R. A. Gosady

**Affiliations:** 1 Department of Laboratory Medicine, Faculty of Applied Medical Sciences, Al-Baha University, Al Aqiq 65779, Saudi Arabia; 2 Molecular Genetics Unit, Alhada Armed Forces Hospital, Taif 26792, Saudi Arabia; 3 Ibn Sina Hospital for Extended Care, Makka 24247, Saudi Arabia; 4 Laboratory Department, King Fahad Hospital Al-Baha, Al Baha 65732, Saudi Arabia; 5 Department of Basic Sciences, Faculty of Applied Medical Sciences, Al-Baha University, Al-Baha 65528, Saudi Arabia; 6 General Hospital of Sabt Al Alaya, Sabt Al Alaya 61985, Saudi Arabia; 7 Laboratory Department Prince Mishari Bin Saud General Hospital, Baljurshi 65622, Saudi Arabia; 8 Laboratory Department, Baish General Hospital, Jazan 87597, Saudi Arabia

**Keywords:** hMPXV, docking, epitope, immunoinformatics, cloning, simulation

## Abstract

**Background**: hMPXV poses a major public health risk due to its human-to-human transmissibility, severe complications, especially in immunocompromised individuals, and global spread, necessitating effective surveillance and stringent prophylactic measures to mitigate its colossal impact. **Objective**: The study aimed to annotate hMPXV(IMV) proteins to propose a potential reverse vaccinology-based vaccine against hMPXV. **Methods**: The target MPXV(IMV) protein’s sequences, formatted in FASTA, were sourced from genome/proteome databases (BV-BRC and UniProt) (accessed on 6 November 2024), followed by CD-Hit-based redundancy removal. Epitope prediction for B-cells (lymphocytes), cytotoxic T-cells or cytotoxic T-lymphocytes (CTLs), and helper T-cells (HTLs) was executed using ABCpred, IEDB’s ANNs 4.0, and an artificial neural network-based alignment tool (NN-align 2.3)/ML-based tool (NetMHCII 2.3). Various immunoinformatics filters (antigenicity, toxicity, and allergenicity) were applied to substantiate the potency and safety of the formulated vaccine candidate. The constructed vaccine’s physiochemical and structural features (secondary and tertiary), with structural stability (confirmed by molecular docking followed by dynamic simulation with TLRs (TLR4 & TLR2) and MHCs), were determined. Additionally, cloning (using pET-28a(+) vector) was conducted to verify the vaccine’s expression potential and translation efficiency. The construct’s population coverage was also ascertained. **Results**: The MPXV-2-Beta vaccine constructs, of the six initially designed constructs, was identified as the most promising candidate, signifying nonallergenic profile and nontoxic features, with a predicted antigenicity score (PAS) = 0.7202, 407 residues, a molecular weight of 43,102.1 Da, pI of 9.2, and favorable stability parameters (AI: 65.65, GRAVY: −0.597, I-i: 25.92). It showed high solubility (score: 0.942). The ProSA Z-score of −9.38 confirmed the structural stability, reliability, and precision of the MPXV-2-Beta 3D model, which is comparable to experimental structures. Furthermore, 98.8% of all the residues nested within favored or allowed regions in a critical Ramachandran plot signified the model’s exceptional structural integrity and quality. Docking and dynamic simulation of MPXV-2-Beta with TLRs (TLR4 & TLR2) and MHCs demonstrated stiffer docking stability (strong polar and nonpolar interaction) and negative eigenvalue value (during dynamic simulation), suggesting its ability to enhance immune receptor activation under physiological conditions. MPXV-2-Beta was predicted to trigger a robust immune response (IR) with comprehensive world population coverage (98.55%, SD = 10.41). **Conclusions**: Based on the evaluated parameters, the MPXV-2-Beta designed in this study exhibited significant potential as an effective candidate against hMPXV. This study establishes a foundation for developing an efficient vaccine against hMPXV, requiring further experimental and clinical validation to confirm computational findings.

## 1. Introduction

The strain of MPXV that causes zoonotic infection in humans, clinically determined by fever, rash, and lesions [1], through multiple transmission routes, including contaminated surfaces [2], inhalation [3], skin contact [4], and sexual contact [5] is known as hMPXV. Furthermore, from recent outbreaks, a new dimension of the transmission pattern has been identified, suggesting that homosexuality (men-to-men) contributes to the rise of hMPXV cases among men [6]. The hMPXV exhibits symptomatic similarity with the variola virus (VARV) [7]; however, the severity of the hMPXV infection is lower than that of the VARV [8]. Monkeypox remained largely confined to countries of the African continent for many years, with only a few sporadic cases reported elsewhere. However, the unanticipated hMPXV spillover into new populations in non-endemic regions has drawn attention [9]. Under the four-month timeframe, MPXV infections escalated beyond 48,000 cases, with 13 confirmed mortalities, accentuating public health concerns [10].

Additionally, a considerable surge of cases in the African population was noticed in 2022 [11]. Between 01/2022 and 10/2023, 91,000 cases caused by hMPXV and new hMPXV variants across various countries (*n* = 115) were reported, based on which the WHO asserted the outbreak (epidemic) to be a global health-related emergency [10,12]. The Poxviridae family encompasses the Orthopoxvirus genus, which includes hMPXV and a symtomatologically similar smallpox virus [13]. Genomic variation has served as a driving force to contribute to the emergence of different clades of hMPXV, including Clade I (virulent and severe), which has high mortality, and Clade II (subclade IIa and subclade IIb), which has lower mortality [14]. First detected in geographical areas outside endemic zones, such as Europe and North America, in 2022, Clade IIb has been identified as the 2022–2024 pandemic strain, with certain mutations that enhance human-to-human transmission. Additionally, the divergence within Clade IIb, with evolving lineages and sub-lineages and with variants/sub-variants that affect human-to-human transmission, symptom severity, reduced effectiveness of the vaccine in use, and therapeutic efficacy, will be a major future challenge in curbing the spread of hMPXV [15,16].

A definitive cure for hMPXV remains unavailable; however, therapeutic interventions and supportive care protocols originally developed for smallpox serve as the principal clinical management strategies for hMPXV infection [17], and vaccines (the second generation ACAM2000 and the third generation JYNNEOS) approved for preventing smallpox are considered protective against hMPXV infection due to cross-reactivity [18,19,20]. Moreover, the declining immune response elicited by earlier hMPXV infection or the protection fostered by the smallpox vaccine [10,21] because of the emergence of the hMPXV lineages and sub-lineages [22] has probably contributed to the surge in cases in recent years. Apart from the effectiveness, the safety of the currently available vaccines is another additional challenge to address because they have been reported to be associated with cardiac side effects [23]. The hMPXV exits in the extracellular enveloped virus (EEV) and IMV forms, exhibiting greater infectiousness [24,25]. Studies have reported that the central genomic region encoding several SPs, which are critical for viral replication and infectivity, is functionally constrained and evolutionarily conserved [26]. Due to their conservation across various hMPXV strains and their functional importance, these proteins have been prioritized as attractive targets for an hMPXV-specific vaccine candidate in many studies [27,28,29,30]. A29L, an IMV envelope protein critical for fusion viral replication [27,31], the antigenic H3L IMV protein responsible for enhanced infectivity [32], the antigenic E8L (a surface membrane protein) of IMV [27], and M1R, a preserved IMV surface protein contributing to viral entry and assembly, have been identified as target proteins in a few studies for therapeutic development, along with other proteins. Applying machine learning (ML)-driven computational immunoinformatics tools, servers, and programs in developing multi-epitope peptide constructs has gained remarkable importance in recent years, particularly during the COVID-19 pandemic [33]. The computational approach facilitates mRNA vaccine design against emerging hMPXV lineages/sub-lineages and validation faster and more cost-effectively. Therefore, the current research aimed to characterize and ascertain epitopes (with antigenicity) for designing and validating an mRNA vaccine construct against lineages/sub-lineages of hMPXV by targeting four antigenic IMV proteins.

This investigation contributes to the foundational knowledge for hMPXV vaccine development; however, it requires experimental and clinical validation of computational predictions. Immunoinformatics and molecular docking enhance design accuracy, while preclinical (in vitro and in vivo) assessments are fundamental to confirming immunogenicity, safety profile, and clinical viability.

## 2. Materials and Methods

**Figure 1:**
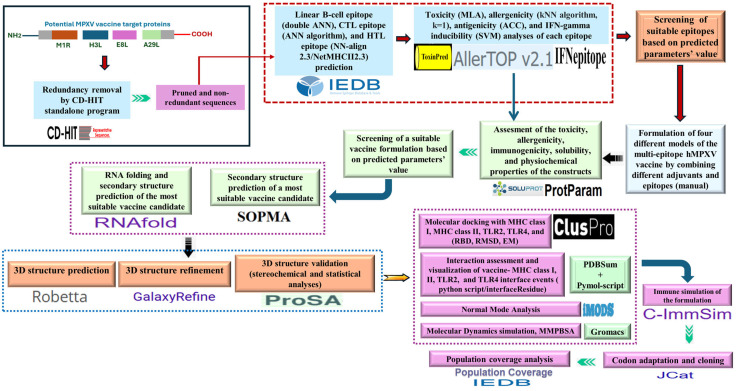
Demonstration of methodological workflow. The details of the server/tools depicted in this figure have been explained under different sections of the methods.

Figure 1 demonstrates a thorough workflow and methodological basis, providing a general idea of the sequential processes and systematic approaches.

### 2.1. Proteins and Their Retrieval, Protein Dataset Generation, and Antigenicity Evaluation

The first step was to retrieve the FASTA-formatted sequences of target IMV proteins (A29L, antigenic H3L, E8L surface membrane protein, and M1R surface protein). These sequences were sourced from the BV-BRC [34] (accessed on 6 November 2024) and Uniprot [35] for further analyses (accessed on 06 November 2024). Four distinct datasets were generated by adding the sequences from two sources and removing the duplicates. The acquired selected protein sequences were evaluated for their antigenic potential using the VaxiJen server. CD-HIT, a redundancy-removing tool, was used to execute the analysis to remove redundancy from the sequences, employing a similarity threshold of 90% to retain non-paralogous target sequences, to reduce the bias within each dataset, and to ensure the reliability for large-scale analysis by using a standalone CD-HIT program [36] (Figure S1 of Supplementary File S1). Subsequently, NCBI BLASTp was used to screen the prioritized proteins against human protein sequences with specific parameters, such as the E-value ≥ 10^−4^, the identity < 50% and query coverage < 50%, to identify the non-paralogous hMPXV-specific protein sequences for downstream processing. The overall characteristics (physical and chemical features, toxicity profile, allergenicity status, expression solubility, and antigenicity) of IMV target proteins were accomplished by employing Expassy/ProtParam (a protein system analysis tool), ToxinPred (toxicity profiling server) [37], AllerTop (allergenic prediction server) [38], soluProt (protein solubility determiner) [39] and VaxiJen (antigenicity predictor) [40] server, respectively.

#### 2.1.1. Antigenic Determinants (Epitopes) Prediction

Predictive analyses were carried out for epitopes (linear B-cell epitopes), which are crucial for an Ab-mediated immune response (Ab-mIR). Immunoinformatically, the prediction of cytotoxic T-lymphocyte (CTL) epitopes was implemented. IEDB tools-based prediction of helper T-lymphocyte (HTL) epitopes, a critical component of cell-mediated IR, was achieved.

#### 2.1.2. Predictive Identification of Linear B-Cell-Epitopes (LBEs)

The prioritized target IMV proteins were processed on the ABCpred to ascertain LBEs [41]. An appropriate cutoff (score = 0.51) was selected to predict B-cell epitopes/antigenic determinants (16-mer). The immunoinformatic server, ABCpred, depends on a neural network algorithm to assess the specificity, sensitivity, accuracy, and positive predictive value for each antigenic LBE [41].

#### 2.1.3. Immunoinformatically Predictive Identification of CTL Epitopes (CTLEs)

The CTLEs were immunoinformatically predicted by harnessing the IEDB consensus methodological strategy with ANN 4.0, which leverages artificial neural networks (ANNs) trained on the data of experimental binding affinity. ANN 4.0 improves its predictions by operating on large, comprehensive datasets, making it significantly effective for recognizing CTLEs [42]. To avoid the sub-optimal immune activation of CD8+ T-cells, only the CTLEs with an IC50 value lower than the restrictive threshold equal to 100 nM were prioritized as principal candidates for incorporation into the proposed vaccine construct.

#### 2.1.4. Computational Prediction of the HTL-Epitopes (HTLEs)

HTLEs were computationally predicted using the neural network-based alignment (NN-align 2.3)/ML-dependent-NetMHCII 2.3 methods accessed from the IEDB platform. High-rank HTLEs were manually screened for vaccine construction. Interferon-gamma (IFN-γ)-inducing HTLEs [43], and IL-4-inducing HTLEs were determined using IFNepitope working on ML-model and IL-4pred, respectively [44].

### 2.2. Comprehensive Evaluation and Prioritisation of Predicted Lbes, Ctles and Htles to Construct an Effective Vaccine

The suitable antigenic determinants (LBEs, CTLEs, and HTLEs) were determined and thoroughly evaluated for their antigenic characteristics (VaxiJen2.0/https://www.ddg-pharmfac.net/vaxijen/VaxiJen/VaxiJen.html) [40]), toxicity status (ToxinPred) [37], soluble expression (SoluProt 1.0) [39] and allergenicity profile (Allertop) [38]. Nontoxic, nonallergenic, and soluble LBEs were screened based on ABCpred scores to construct an effective vaccine. CTLEs and HTLEs exhibiting appropriate features (nontoxic, nonallergenic, and soluble) were determined based on their antigenicity scores from VaxiJen 2.0 for adding to the construct.

### 2.3. Chimeric Vaccine Model Design and the Screening of an Effective Vaccine Model

Immune epitopes, including those for B-cells, HTLs, and CTLs, were strategically merged using specialized amino acid linkers to produce two (*n* = 02) chimeric hMPXV vaccine prototypes. These two prototypes were conjugated separately with three types of adjuvants: one set each with mammalian beta-defensin and heparin-binding hemagglutinin adhesin (Hbha) and the other with ribosomal protein, producing the six (*n* = 06) prototypes eventually. The inclusion of specific linkers to join the epitopes for optimizing protein expression and ensuring a robust and sustained immunogenic response was accomplished. LBEs were connected using GPGPG linkers, HTLEs were joined with KK linkers, and AAY linkers were used for CTLEs [45]. Subsequently, all six vaccine prototypes were assessed for their antigenic and physiochemical properties. For optimal effectiveness and safety, the vaccine must exhibit stability, non-allergenicity, antigenicity, nontoxicity and high solubility. Therefore, the physicochemical attributes, antigenic potential, allergenic profile, toxicity, and expression solubility of all six vaccine prototypes were meticulously assessed using ProtParam (https://web.expasy.org/protparam/), VaxiJen v2.0 [40], AllerTop [38], ToxinPred, SoluProt 1.0 [39], respectively. Based on these properties (antigenicity status, solubility, toxicity and physiochemical characteristics), the most appropriate vaccine prototype (*n* = 01) of six was screened for further analyses. MPXV-2-Beta, compared with MPXV-1-Beta, MPXV-1-Hbha, MPXV-1-Ribos, MPXV-2-Hbha, MPXV-2-Ribos was selected as the most suitable candidate.

Furthermore, a well-known, non-natural, pan-DR epitope for activating HTL was added to the vaccine prototype to strengthen the vaccine’s potency and effectiveness. In addition to other elements, the Kozak sequence comprising the start codon has been recognized as one of the most crucial elements of the mRNA vaccine. In addition to the Kozak sequence, a well-constructed mRNA vaccine typically requires antigenic determinants, an appropriate adjuvant, and functional linkers to get optimum translational efficiency [46]. However, the stop codon could be enhanced [47]. A signal peptide (at N-terminus) aiding the extracellular secretion of translated epitopes and an MHC-class-I-trafficking-signal (MITD) linked to the terminus (C-terminus) of the antigenic protein represent two critical elements for the optimum efficiency of the contract. MITD is required to ensure antigen presentation via the MHC-I pathway. Therefore, the 5′ region of the open reading frame (ORF) was designed to include the tissue plasminogen activator (tPA) signal peptide obtained from UniProt (ID: P00750) at the N-terminus of the antigen-coding sequence.

Additionally, the MHC class I trafficking domain (MITD) sequence, obtained from UniProt (ID: Q8WV92), was appended to the C-terminus of the antigenic construct within the 3′ region of the ORF [48,49]. The instability of the transcript of mRNA-based vaccines poses an additional challenge, and to address the issue, elements normally found in eukaryotic mRNAs must be incorporated [50,51]. To address the mRNA instability issue, the vaccine prototype was designed by incorporating four essential components (5′ m7G cap, poly(A) tail, 5′ untranslated regions (UTRs) and 3′ UTRs). These critical elements were integrated to improve mRNA stability and translational efficiency [52]. Additionally, standardizing the segment length of the poly(A) tail is critical because excessively short or overly long tails affect protein translation efficiency [53]. Typically, an optimal poly(A) tail length of approximately 115–150 nucleotides is considered suitable for the mRNA vaccine efficacy [54]. Two other significant components were incorporated; NCA-7d was integrated into the 5′ UTR to stabilize the mRNA structure, while S27a+R3U was added into the 3′ UTR [55,56].

### 2.4. Vaccine’s Secondary Level Structure Prediction

The computational prediction of the secondary structural composition: beta-sheets, coil and alpha-helices of the selected vaccine prototype was executed using the SOPMA [57], and the PSIPRED 4.0 server [58]. These servers analyze key structural elements, for instance, transmembrane helices, topology, and folding patterns within a given peptide sequence, to provide an understanding of the vaccine prototype stability and functional features. The mRNA secondary structural feature analysis was executed employing the RNAfold of ViennaRNA (v. 2.0) [59], which makes a prediction of the centroid secondary mRNA structure and computes McCaskill’s algorithm-based estimation of the allied metrics, for instance, the minimal free energy (MFE) [60].

### 2.5. Three-Dimensional Structural Modelling and Computational Refinement of the Most Suitable Vaccine Prototype

The three-dimensional structure of a protein or peptide offers comprehensive insights into its structural stability, functional features, and interactive potential with other biomolecular structures. The three-dimensional structure of the most suitable vaccine prototype was produced by employing the Robetta server, which ensures accurate and reliable structural insights leading to the evaluation of the vaccine constructs’ stability, folding, and functionality [61]. Another server, Galaxy Refine, was used for further refinement of the 3D structure by getting the .pdb file input, which was produced by the Robetta server [62]. After refining and enhancing structural features, the model’s structure quality was subsequently optimized by the PROCHECK server. Furthermore, the ProSA server-dependent validation of the prototype was accomplished [63].

### 2.6. Molecular Docking and Normal Mode Analysis (Nma) for Gaining Analytical Insight into Molecular Complexes

To successfully trigger a sustained, robust, and targeted host IR, the vaccine must exhibit precise and conformationally stable interactions with key immune receptors to foster efficient recognition and activation of immunological pathways. The files, in .pdb format and of the toll-like receptors TLR4 (PDB Id; 4G8A) and TLR2 (PDB Id; 2Z7X), were acquired via the protein data bank (PDB). The files, in .pdb format, of MHC class I (PDB Id-2XPG) and MHC class II (PDB Id-1KG0) were also acquired from the same PDB database. Pymol-based preprocessing (identification and manipulation of the chain identifier) was executed on these TLRs and MHCs .pdb files. Following the preprocessing of the files, ClusPro 2.0-assisted receptors’ docking with the vaccine prototype was executed [64]. Subsequently, PDBsum (web-based bioinformatics tool), in conjunction with the application of Pymol script/commands, was harnessed to evaluate interaction events (residues and atomic-level biochemical interactions) at the interface of individual complexes [65]. The iMODS server, a tool designed for the normal mode analysis (NMA) of biomolecular structures, especially protein complexes, was utilized to evaluate the intrinsic dynamic behavior, flexibility, and stability of the molecular structures of the TLR-vaccine and MHCI–vaccine complexes with the default parameters of the server. iMODS provides insights into molecular motions and flexibility by evaluating the intrinsic vibrational modes of the complexes [59]. iMODS enables the assessment of biomolecular complex dynamics by performing energy minimization and simulating atomic and molecular movements to reveal their structural flexibility and intrinsic motion patterns. [66].

### 2.7. Molecular Dynamics Simulation (Mds) of the Tlr-Receptors (TLR4 and TLR2)-Vaccine Prototype Complexes

The Gromacs bioinformatics tool was leveraged to execute molecular dynamics simulations (MDS) of vaccine prototypes and TLR–vaccine complexes for 100 ns to determine the stability of the vaccine and vaccine complexes with immune receptors in an aqueous environment, thereby gaining insights into the binding interactions of the vaccine with receptors. The simulation system was prepared and parameterized by employing the AMBER99SB force field [67]. The complex solvation was achieved in a triclinic simulation box filled with explicit water molecules (simple point charge (SPC) water model) to mimic the aqueous environment. Neutralization was achieved by the physiological concentration of Na + or Cl- ions (0.15 M) to maintain ionic strength and charge neutrality. Energy minimization (EM steps = 5000, EM-integrator = steepest descent algorithm) was performed to relax the initial configuration of the TLR–vaccine complexes. The system was equilibrated to attain thermodynamic equilibrium (at 310 K and 1 bar pressure), with a time step of 100 picoseconds (ps) under NVT ensembles, followed by NPT ensembles [68]. Temperature coupling was applied to maintain a stable thermal environment, while pressure coupling was used to allow the simulation box to adjust its volume in response to the target pressure, ensuring thermodynamic stability. Following the equilibration process, production MDS was undertaken for a total duration of 100 nanoseconds (ns). A post-simulation study was carried out to assess the root mean square deviation (RMSD), root mean square fluctuation (RMSF), the number of hydrogen bonds, and gyration radius (Rg) on the MDS trajectory obtained through Gromacs [69]. Moreover, energy calculations were also performed to gain a deeper insight into the intermolecular strength between the vaccine and TLRs. Furthermore, post-MDS, the molecular mechanics Poisson–Boltzmann surface area (MMPBSA), encompassing metrics for the stability and interaction strength of biomolecular complexes, was leveraged to estimate the binding free energy (ΔGbind) between the vaccine prototype and the TLRs and immune molecules for 100 ns and also for the last 20 ns of simulation [70]. Additionally, Gromacs potential energy, kinetic energy, and short-range electrostatic (Coulombic) energy for TLR–vaccine complexes were also analyzed.

### 2.8. Implementation of Codon Adaptation and Cloning of Vaccine Prototype with an Expression Vector

The vaccine prototype’s protein sequence was subjected to a web-based server/tool for reverse translation. After reverse translation, the DNA sequence of the prioritized prototype was processed for codon optimization, utilizing Java-Codon-Adaptation Tool (JCAT) algorithms to enhance and evaluate the construct’s translational yield and expression efficiency [71]. The GC content (in DNA) and codon adaptation index (CAI) were substantiated. For the CIA; 1.0 was considered an ideal value, while scores surpassing 0.8 were considered optimum for robust expression. The GC content (most favorable within a range of 30% to 70%) was also determined, as deviations outside this range may undesirably influence transcriptional and translational processes, thus impacting protein expression [72]. pET28a(+) (expression vector) and the *E. coli* expression system acquired from SnapGene tool (SnapGene|Software for everyday molecular biology) were leveraged to perform cloning of the critically optimized (by JCAT) vaccine prototype sequence [73]. However, there is a limitation with codon optimization in prokaryotic systems. As the current research is an in silico study, further experimental validation using a eukaryotic expression system, such as HEK293 cells or in vitro cell-free human translation systems, will be required to confirm translational efficacy in the human host system.

### 2.9. Immune Response Simulation

The immunogenic potential of the screened, docked, and promising vaccine prototype was assessed through computational immuno-simulation approaches with the aid of the C-ImmSim server/tool. This platform integrates position-specific scoring matrices (PSSM) for precise computational prediction of IRs. The C-ImmSim server functions at a cellular-level, as an agent-based immunoinformatics algorithm that accurately simulates the intricate interactions within the mammalian immune system. It specifies detailed features of the IRs elicited by antigens (vaccine prototype). The server systematically evaluates the IRs to the proposed vaccine prototype [74]. The simulation was conducted for the initial dose (at zero days) and was followed by two booster doses (a booster dose administered at 30 days and 60 days). All parameters, including random speed and MHC class I and II alleles, were kept as default. The parameters were configured with a volume setting 10, and the effect was assessed for 300 days.

### 2.10. Population Coverage Assessment

Moreover, population coverage assessment (by considering alleles of both classes of MHC) was accomplished by leveraging the IEDB analysis resource [75]. Based on HLA allele distribution, the population coverage resource integrated with IEDB evaluates the percentage (proportion) of individuals within a particular population that could respond to a provided set of epitopes (CTLEs and HTLEs). A population coverage tool, which operates at standard parameters (default parameters), was employed to perform the analysis.

## 3. Results

### 3.1. IMV Target Sequence Retrieval and Processing for Their Suitability for Vaccine Development

Four IMV target proteins, A29L (no. of sequences = 638), H3L (no. of sequences = 576), E8L (no. of sequences = 569), and M1R (no. of sequences = 563) were processed (accessed on 6 November 2024). The accession numbers (ANs) of the four selected virus-specific non-paralogous protein sequences for downstream study are as follows: A29L (AN: QJQ40281.1 A29L), H3L (AN: AGR38652.1), E8L (AN: QJQ40248.1) and M1R (AN: QJQ40223.1) (Figure S1 of Supplementary File S1). These four IMV proteins were processed to predict potential LBEs, CTLEs, and HTLEs. Moreover, the physical and chemical assessment of the target proteins unraveled the hydrophilic nature of the proteins (GRAVY <0). M1R/IMV (Grand Average of Hydropathy (GRAVY) = −0.004), E8L/IMV (GRAVY = −0.359), A29L/IMV (GRAVY = −0.75) and H3L/IMV (GRAVY = −0.02). All four (*n* = 04) target proteins were assessed to be nontoxic and non-allergenic, which is critical for a protein to be a candidate (Table 1). The antigenicity score, solubility, AA chain length, theoretical pI and other physiochemical matrices of all of the proteins are summarized in Table 1.

### 3.2. LBE Determination and Incorporation into Vaccine Construct

LBEs, key elements for vaccine effectiveness, were determined. The most promising LBEs were determined based on their antigenicity score, which reflects their strong interaction with B-cell receptors, which is a significant factor in activating humoral IR. The criteria for screening of LBEs included multiple factors, such as high binding ABCpred scores, antigenicity (exceeding a threshold/cutoff of 0.4), nontoxic nature, and nonallergenicity. Eight (*n* = 08) LBEs (*n* = 02 from each protein) were determined for incorporation into the final vaccine constructs. The binding affinity (ABCpred scores), antigenicity scores, toxicity status and allergenicity of all of the LBEs are summarized in Table 2.

### 3.3. Evaluation of CTLEs for Incorporation into Vaccine Construct

CTLEs were assessed because CTLs/CD8+ T cells are pivotal in defending against viral infections. The CTLEs with IC_50_ value < 100 nm were prioritized for further evaluation. The CTLEs with nontoxicity, nonallergenicity and positive antigenicity values (top-ranked; score > 0.5) were carefully chosen to incorporate into the vaccine construct. Eight (*n* = 08) CTLEs (*n* = 02 from each target IMV protein) were picked for integration into the final vaccine construction. The CTLE–allele pair, IC_50_ value, antigenicity scores, toxicity status and allergenicity profile are outlined in Table 2.

### 3.4. Determination of Potential Htles for Incorporation into Vaccine Construct

This research evaluated 15-mer HTLEs with their MHC class II alleles pair using the MHC-II binding assessment tool (present on IEDB). The HTLEs with an IC_50_ value < 100 nm were prioritized. The nontoxic, nonallergenic, IL-4-inducing HTLEs with appropriate antigenicity values (high-ranked, with a score > 0.5) were chosen for incorporation into the vaccine construct. Eight (*n* = 08) HTLEs (*n* = 02, retrieved from individual proteins) were picked for incorporation into vaccine formulation. Additionally, two (*n* = 02) from A29L/IMV-based high score (0.9657 and 0.6529) IFN-γ-inducing HTLEs with IL-4-inducing overlapping activity (IL-4pred score = 0.31) were screened for inclusion in vaccine constructs. Table 3 illustrates the HTLE–allele pair, IC50 values, antigenicity scores, IL-4pred scores, IL-4-inducing potentials and IFN-γ-inducing toxicity and allergenicity statuses of all of the the HTLEs.

### 3.5. Chimeric Vaccine Design and Screening for the Potential Vaccine Model

Two (*n* = 2) vaccine constructs were initially designed by integrating selected linear B-cell epitopes (LBEs), cytotoxic T-lymphocyte epitopes (CTLEs), helper T-lymphocyte epitopes (HTLEs), and two (*n* = 2) IFN-γ-inducing epitopes derived from the A29L/IMV protein. These components were combined using specific linkers appropriate for each epitope type. Subsequently, three different adjuvants (heparin-binding hemagglutinin (Hbha), mammalian beta-defensin, and ribosomal protein) were individually incorporated into the initial constructs, resulting in six (*n* = 6) final vaccine constructs (MPXV-1-Beta, MPXV-2-Beta, MPXV-1-Hbha, MPXV-1-Ribos, MPXV-2-Hbha, MPXV-2-Ribos), which were subjected to further immunoinformatics analysis. The adjuvants were attached via a linker (EAAAK) to the terminal (N-terminal) of the designed prototype sequences. The amino acid (AA) length/molecular weights of the MPXV-1-Beta, MPXV-2-Beta, MPXV-1-Hbha, MPXV-1-Ribos, MPXV-2-Hbha, MPXV-2-Ribos were 405/42,760.94, 407/43,102.1, 519/55,228.39, 490/51,040.22, 521/55,569.55 and 492/51,381.39, respectively (Table 4). All six (*n* = 06) constructs were antigenic, nontoxic, and nonallergenic. However, the antigenicity score (0.7495) for construct MPXV-1-Beta was the highest, followed by MPXV-2-Beta (0.7202). Moreover, the expression solubility (Soluproscore = 0.942, cut off ≥0.5) for MPXV-2-Beta was the highest, followed by MPXV-1-Beta (Soluproscore = 0.836, cut off ≥0.5), as mentioned in Table 4. As the antigenicity score of MPXV-2-Beta was comparable to that of the MPXV-1-Beta, however, expression solubility (Soluproscore = 0.942) for MPXV-2-Beta was comparatively higher than MPXV-1-Beta. Therefore, the MPXV-2-Beta vaccine prototype was assessed as the most potential prototype for downstream analysis. The theoretical pI (TpI), aliphatic index (AI), GRAVY, and instability index (I-i) of MPXV-2-Beta were assessed to be 9.77, 65.63, −0.597 and 25.92, respectively. A negative GRAVY score (cut off = zero) for selected MPXV-2-Beta demonstrated that the prototype was potentially hydrophilic and comparatively highly soluble. An Ii score of 25.92 of the MPXV-2-Beta suggested that the construct was stable and soluble. Additionally, the aliphatic index (65.63) between 40–85 of the chosen MPXV-2-Beta construct demonstrated its appropriate thermostability and solubility. The details of physiochemical parameters, antigenicity scores, toxicity scores, solubility scores, half-life period and allergenicity status for all six (*n* = 06) constructs are mentioned in Table 4.

### 3.6. Prediction of the Secondary for Screened Chimeric Vaccine Prototype (MPXV-2-Beta)

**Figure 2:**
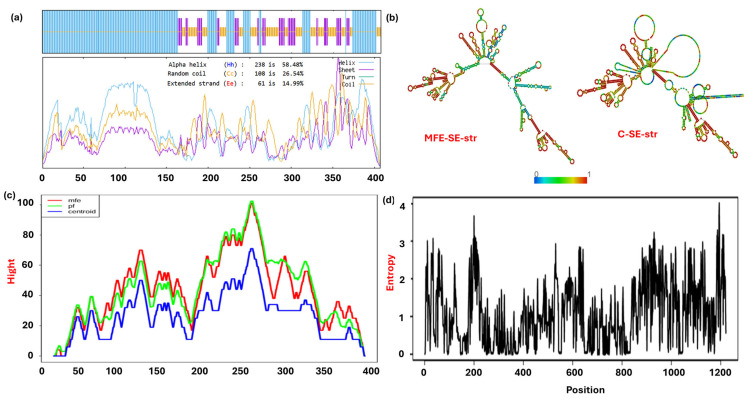
The predicted structures (secondary structure of protein and mRNA) of the screened vaccine prototypes. (**a**) The helix, sheet, turns, and coils (present in the secondary protein structure) of the construct (MPV-2-Beta), (**b**) mRNA secondary structure (MFE-SE-str = mean free energy secondary structure and c-SE-str = centroid secondary structure), (**c**) mountain plot representing the mean free energy, thermodynamic ensemble, and centroid structure of mRNA, and (**d**) entropy plot illustrating positional entropy for each position in mRNA.

The predicted secondary structures of the MPXV-2-Beta vaccine demonstrated that the prototype was composed of 58.48% α-helices, 14.99% extended strands, and 26.54% random coils, as depicted in Figure 2a and Figure S2 of Supplementary File S1. The secondary mRNA structure was thermodynamically stable, with a very low (negative value) minimum-free-energy (MFE) of −454.4 kcal/mol and free energy of thermodynamic-ensemble (TDE) (−479.13 kcal/mol) with moderate ensemble diversity (305.27). The centroid secondary structure, representing the ensemble’s most probable or representative structure, showed an MFE of −333.2 kcal/mol, as illustrated by dot-bracket annotation (Figure S3 of Supplementary File S1). Figure 2b demonstrates only a minor region with high entropy (region of high variability), whereas other regions are blue to green (representing low entropy and higher stability). The mountain plot exhibits most of the area where lines overlap, suggesting a strong agreement between the MFE, partition function (PF), and centroid predictions and a stable and distinct structure in those regions, as depicted in Figure 2c. Entropy is a significant factor for RNA secondary structure prediction; low entropy values signify positions in the RNA sequence where the predicted structure shows minimal variability. The entropy graph exhibits a positional entropy value of less than 2.0 at most positions, suggesting stable and distinct mRNA secondary structures. However, the entropy value is slightly more than 2.0 at a few positions, explaining the presence of flexible and less stable regions (Figure 2d).

### 3.7. Prediction of 3D Structures for Chimeric Vaccine Construct (MPXV-2-Beta)

**Figure 3:**
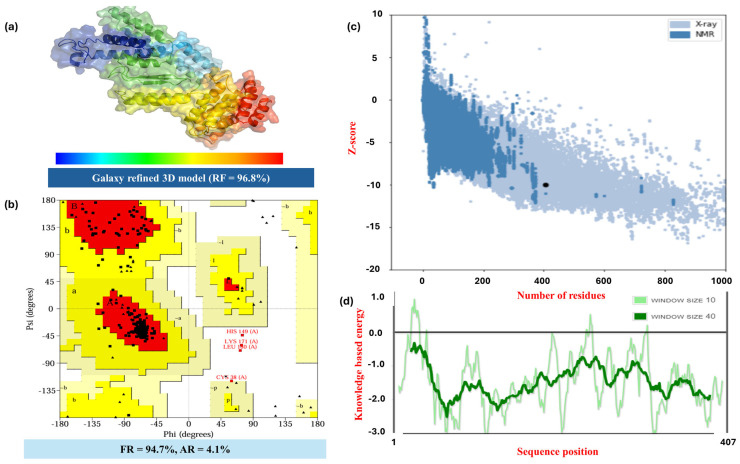
The illustration of the three-dimensional (3D) structure of the screened construct (MPXV-2-Beta) and validation of the 3D structure: (**a**) Robetta-predicted and Galaxy-refined 3D model of the MPXV-2-Beta construct (tertiary structure of the MPXV-2-Beta multi-epitope vaccine construct rendered in cartoon representation and colored using a rainbow spectrum to illustrate residue progression from the N-terminal (blue, left) to the C-terminal (red, right)), (**b**) Ramachandran plot, (**c**) Prosa-web-based validation plot with z-score, and (**d**) energy validation plot of a 3D model of the MPXV-2-Beta construct. RF = Ramachandran favored, FR = favored region, and AR = allowed region. Black dot represents the structure of interest (structure under evaluation). Most residues fall within the favored (red) and allowed (yellow) regions, indicating good stereochemical quality. A few residues—HIS149(A), LYS171(A), LEU180(A), and CYS38(A)—appear in the disallowed regions.

GalaxyRefine processed and refined the 3D structure of MPXV-2-Beta. The top ranked GalaxyRefine model (Rama favored 96.8%), visualized by Pymol, is depicted in Figure 3a. The computed ProSA (Z-score) was approximately −9.38 for the 3D model of MPXV-2-Beta, which demonstrates its structural stability and reliability. Furthermore, the position of the query prototype in the graph demonstrates that the model’s structural quality resembles the reliable experimentally validated structures of the vaccine (Figure 3b). The protein structure was further authenticated by analyzing backbone dihedral angles of amino acid residues. Phi (Φ) and psi (Ψ) were the two specific dihedral angles under analysis. The Ramachandran plot indicated 98.8% of all the residues, comprising 94.7% in favored regions (FRs) plus 4.1 % in allowed regions, suggesting a high-quality 3D model of MPXV-2-Beta (Figure 3c). However, a small percentage of residues fell in the disallowed areas (1.2%) (possibly because of the flexible loops or special conformations), which is predictable and acceptable within limits. Moreover, the stability of the structure was authenticated through additional validation by knowledge-based energy as a function of sequence position (Figure 3d). Knowledge-based energy plots demonstrate that most sequences have negative energy scores, suggesting that the 3D structure is generally stable in regions with stronger negative peaks. The structural validation results reveal substantial stability of the MPXV-2-Beta structure, rendering it suitable for further docking analysis.

### 3.8. Computational Docking of MPXV-2-Beta Interactions with TLRs and Major Histocompatibility Complex Molecules (MHC Molecules)

**Figure 4:**
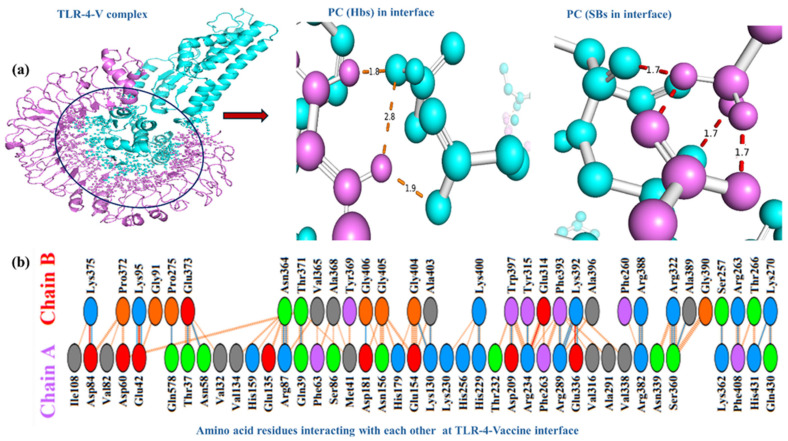
TLR-4–MPXV-2-Beta vaccine interaction. (**a**) TLR-4–MPXV-2-Beta interactions (violet color represents TLR4 chain, cyan color denotes the MPXV-2-Beta chain, and the black circle represents the interface), PC = polar contacts, Hbs = hydrogen bonds, SBs = salt bridges. (**b**) The amino acid (AA)-residues interacting in the TLR-4–MPXV-2-Beta interface (chain A = TLR4 and chain B = MPXV-2-Beta).

Docking analysis involving MHCs with the proposed chimeric MPXV-2-Beta vaccine are essential for evaluating its immunogenic potential. The MPXV-2-Beta construct was precisely docked with TLR4 and TLR2 for determining the receptor binding dynamics. Figure 4a illustrates the molecular interaction interface and interface events (at residue and atomic levels) of the TLR-4–MPXV-2-Beta vaccine complex with polar contact in the interface (hydrogen bonds and salt bridges with their bond lengths). Figure 4b depicts the type of amino acid residues of TLR4 interacting with residues of the MPXV-2-Beta vaccine. There were 41 residues of TL4 and 31 residues of MPXV-2-Beta vaccine interacting, involving *n* = 04 salt bridges, *n* = 28 hydrogen bonds and *n* = 206 non-bounded contacts with a total interface of 1709–1936 Å^2^, suggesting strong rigid docking interaction (Table 5).

**Figure 5:**
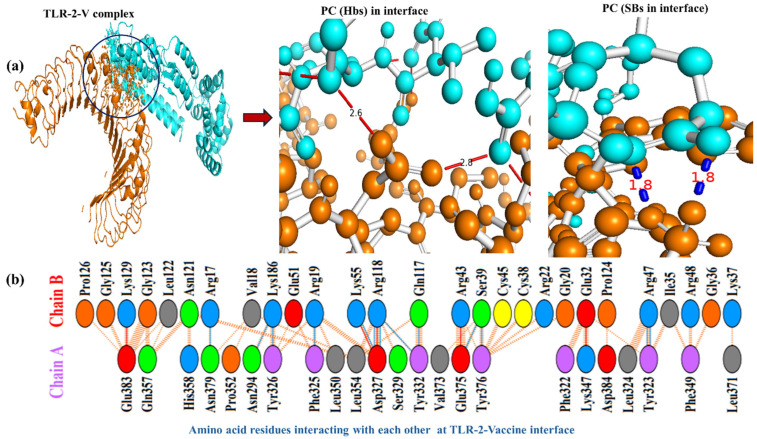
TLR-2–MPXV-2-Beta vaccine interaction. (**a**) TLR-2–MPXV-2-Beta interactions (violet color represents TLR2 chain, cyan color denotes the MPXV-2-Beta chain, and the black circle represents interface), PC = polar contacts, Hbs = hydrogen-bonds, SBs = salt bridges. (**b**) The amino acid (AA)-residues interacting in the TLR-2–MPXV-2-Beta interface (chain A = TLR2 and chain B = MPXV-2-Beta).

Figure 5a illustrates the molecular interaction interface and interface events (at residue and atomic level) of the TLR-2–MPXV-2-Beta vaccine complex with polar contact in the interface (hydrogen bonds and salt bridges with their bond lengths). Figure 5b depicts the type of amino acid residues of TLR2 that interact with the residues of the MPXV-2-Beta vaccine. There were 23 residues of TL2 and 20 residues of MPXV-2 vaccine interacting, involving *n* = 05 salt bridges, *n* = 13 hydrogen bonds and *n* = 159 non-bounded contacts with a total interface of 1112–1030 Å^2^, suggesting stable docking interaction (Table 5).

**Figure 6:**
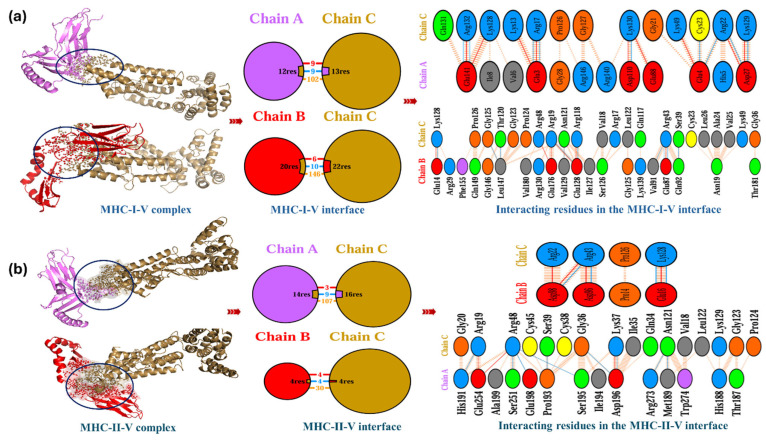
Illustration of MHC–MPXV-2-Beta vaccine interaction. (**a**) MHC class-1–MPXV-2-Beta interactions (sand color chain C represents MPXV-2-Beta, violet color chain A denotes chain A of the MHC class-I molecule, red color chain B denotes chain B of the MHC class-I molecule, and the black circle represents the interface between MHC class-I and MPXV-2-Beta). (**b**) MHC class-II–MPXV-2-Beta interactions (sand color chain C represents MPXV-2-Beta, violet color chain A denotes the chain A of the MHC class-II molecule, red color chain B denotes the chain B of the MHC class-II molecule, and the black circle represents an interface between MHC class-II and MPXV-2-Beta).

Figure 6a illustrates the interaction between MHC class I and the proposed MPXV-2-Beta vaccine (MHC class I–MPXV-2-Beta interface, number of AA-residues of MPXV-2-Beta and MHC class I involved in the interaction at interface, and number of polar and nonpolar contacts along with the types of amino acid residues involved). Figure 6b demonstrates the interaction between MHC class II and the proposed MPXV-2-Beta vaccine (MHC class II–MPXV-2-Beta interface, number of amino acid residues of MPXV-2-Beta and MHC class II involved in the interaction at interface, and number of polar and nonpolar contacts along with the types of amino acid residues involved). A total (chain A and chain B) number (*n* = 32) of MHC class I residues and *n* = 35 residues of MPXV-2-Beta vaccine interacted, involving *n* = 15 salt bridges, *n* = 19 hydrogen bonds and *n* = 248 non-bounded contacts (Table 5). A total (chain A and chain B) number (*n* = 18) of MHC class II residues and *n* = 22 residues of MPXV-2-Beta vaccine interacted, involving *n* = 7 salt bridges, *n* = 13 hydrogen bonds and *n* = 137 non-bounded contacts (Table 5), indicating the unraveled but stable and rigid binding of MPXV-2-Beta with MHC molecules.

### 3.9. Normal Mode Analysis (NMA) and Molecular Dynamic Simulation (MDS) of MPXV-2-Beta with TLRs and MHC Molecules

Stability, flexibility, and dynamic behavioral analysis of TLR4–MPXV-2-Beta, TLR2–MPXV-2-Beta, MHC-I–MPXV-2-Beta, and MHC-II–MPXV-2-Beta were undertaken by employing NMA. The principal deformation plot for the TLR-4–MPXV-2-Beta complex indicated that most regions displayed low flexibility, indicating a stable conformation. Larger peaks at sequence positions underscore the regions of higher flexibility, which could correspond to functional or dynamic regions like binding sites, loops, or hinges. The deformity graph revealed that most of the structure is stable and rigid, with a few localized regions showing higher flexibility. The flexible region may be crucial for dynamic interactions, such as binding or conformational changes.

**Figure 7:**
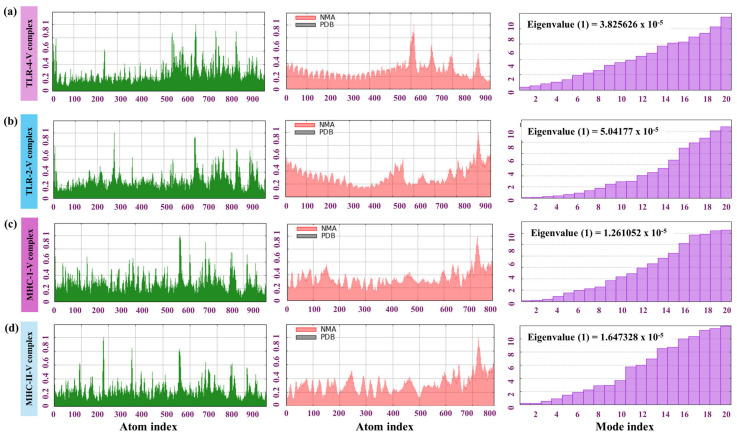
NMA of TLR–MPXV-2-Beta and MHC–MPXV-2-Beta. (**a**) NMA results of TLR4–MPXV-2-Beta, (**b**) NMA results of TLR2–MPXV-2-Beta, (**c**) NMA results of MHC class I–MPXV-2-Beta and (**d**) NMA results of MHC class II–MPXV-2-Beta complexes. Left-side plot: beta factor/mobility graph (flexibility/deformity value vs atomic index); middle plot: comparison between the normal mode analysis (NMA) and PDB-derived flexibility data for a protein or molecular complex. The overlay of NMA (red) and PDB (black) provides insight into the consistency between computational and experimentally derived structural flexibility and right-side plot eigenvalues obtained from normal mode analysis (NMA), plotted against the mode indices.

Furthermore, the eigenvalue (0.3825626 × 10^−4^) for the TLR4–MPXV-2-Beta distribution suggests a balance between flexibility and stability, which is crucial for biological activity and structural integrity as the low eigenvalue (negative) at early mode indicates that the structure is allowed for significant collective motions, essential for functional flexibility (binding interactions or conformational changes) (Figure 7a). However, the higher eigenvalue in later modes indicates the localized rigidity, which could correspond to stable structural regions (Figure 7a). Similarly, the eigenvalues of TLR2–MPXV-2-Beta, MH C class I-MPXV-2-Beta and MHC class II–MPXV-2-Beta (0.50417 × 10^−5^), (0.1261 × 10^−4^), and (0.1647 × 10^−4^) as illustrated in Figure 7b–d demonstrated the appropriate structural stability of the docked complexes. Moreover, the deformity plot for TLR2–MPXV-2-Beta, MH C class I-MPXV-2-Beta, and MHC class II–MPXV-2-Beta indicated that the majority of the structure was stable and rigid, with a few localized regions exhibiting higher flexibility Figure 7b–d. Overall, the NMA results of the TLR–MPXV-2-Beta complexes showed the required flexibility suitable for the biological activity of the complex. The NMA for the complexes (MHC-class-I-MPXV-2-Beta and MHC-class-II-MPXV-2-Beta) suggested appropriate flexibility and functional interactions.

**Figure 8:**
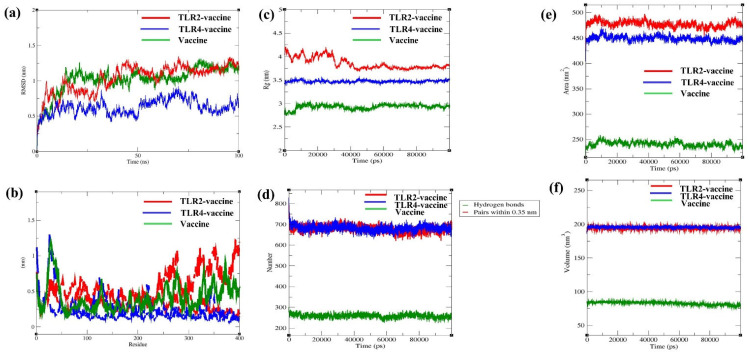
Illustration of RMSD, RMSF, radius of gyration, hydrogen bond formation, SAS area, SAS volume of the vaccine, TLR2 vaccine, TLR4 vaccine. (**a**) RMSD, (**b**) RMSF, (**c**) radius of gyration, (**d**) hydrogen bond formation, (**e**) total solvent accessible area, and (**f**) surface accessible volume.

After an initial RMSD rise (indicating conformational adjustment), the trajectory flattens after ~20–30 ns of simulation with a limited-range deviation (vaccine: ~1.0–1.4 nm, TLR2 vaccine: ~1.0–1.4 nm, and TLR4 vaccine: ~0.4 and ~0.8 nm) without large deviation or drift (Figure 8a). RMSF profiles indicate localized flexibility in the N- and the C-terminal with fluctuation values ranging from ~0.2 to 1.6 nm. The deviation remained largely within a stable range (vaccine: ~0.2–1.2 nm, TLR2 vaccine: ~0.3–1.6 nm, and TLR4 vaccine: ~0.2–0.8 nm) without signs of excessive residue-level instability (Figure 8b). Following earlier fluctuations, the Rg of all complexes stabilized in the early phase of simulation, maintaining compactness with limited-range variation (vaccine: ~2.8–3.1 nm, TLR2 vaccine: ~3.8–4.2 nm, and TLR4 vaccine: ~3.3–3.6 nm) without significant structural expansion or collapse throughout the 100 ns simulation (Figure 8c). The hydrogen bond trajectories remained consistent over the simulation period, exhibiting stable intermolecular interactions (vaccine: ~250–290 bonds, TLR2 vaccine: ~650–720 bonds, and TLR4 vaccine: ~680–740 bonds), which indicates the sustained hydrogen bonding and structural integrity in the receptor–vaccine complexes (Figure 8d). Additionally, the stable solvent-accessible surface area (Figure 8e) and volume (Figure 8f) throughout the simulation suggests the structural compactness and conformational stability of the complex without significant unfolding, expansion, or collapse.

Moreover, MMPSA energy for 100 ns and the last 20 ns were computed to assess the binding energy of TLRs (TLR2 and TLR4)–vaccine complexes. Binding free energy (ΔGbind) estimated for the last 20 ns and full 100 ns of the simulation of the TLR2 vaccine was −386 kJ/mol or −92.40 kcal/mol (complex: −22215.97 kcal/mol, TLR2: −13933.30 kcal/mol and vaccine: −8190.28 kcal/mol) and −339.23 kJ/mole or −81.08 kcal/mol (complex: −22185.63 kcal/mol, TLR2: −13,928.54 kcal/mol and vaccine: −8176.01 kcal/mol), respectively.

ΔGbind computed for the last 20 ns and full 100 ns of simulation of the TLR4 vaccine was −503.8 kJ/mol or −120.42 kcal/mol (complex: −21,734.38 kcal/mol, TLR4: −13,448.83 kcal/mol, and vaccine: −8165.14 kcal/mol) and −454.2 kJ/mole or −108.55 kcal/mol (complex: −21,703.30 kcal/mol, TLR4: −13,435.57 kcal/mol, and vaccine: −8159.19 kcal/mol), respectively. A comparative assessment of ΔGbind across the full 100 ns and the final 20 ns of the TLR2/TLR4 vaccine simulation indicates that the complex maintained a stable interaction profile throughout the trajectory, with a slight increase in the last phase of the simulation.

**Figure 9:**
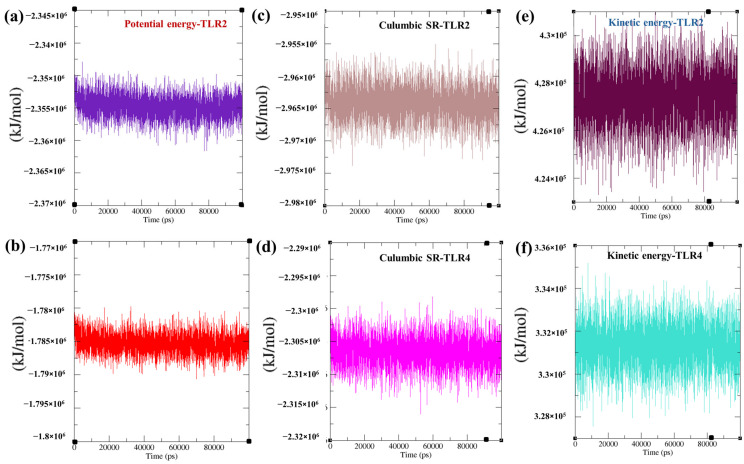
Demonstration of potential energy, short-range electrostatic energy, and kinetic energy of TLR2 vaccine and TLR4 vaccine. (**a**) Potential energy of the TLR2 vaccine, (**b**) potential energy of the TLR4 vaccine, (**c**) short-range electrostatic energy of the TLR2 vaccine, (**d**) short-range electrostatic energy of the TLR4 vaccine, (**e**) kinetic energy of the TLR2 vaccine, and (**f**) short-range electrostatic energy of the TLR4 vaccine.

Furthermore, total potential energy (TLR2:~ −2.355 × 10^6^ kJ/mol (Figure 9a) and TLR4: −1.785 × 10^6^ kJ/mol (Figure 9b)), short-range electrostatic energy (TLR2: ~−2.965 × 10^6^ kJ/mol (Figure 9c) and TLR4:~ −2.305 × 10^6^ kJ/mol (Figure 9d)) and kinetic energy (TLR2: ~ 4.28 × 10^5^ kJ/mol (Figure 9e) and TLR4: ~3.32 × 10^5^ kJ/mol (Figure 9f)) over 100 ns simulation indicated structural integrity, strong electrostatic stabilization, and thermal stability of the TLR–vaccine complexes, respectively.

### 3.10. Assessment of the Potency of the MPXV-2-Beta by Immunological Simulation

**Figure 10:**
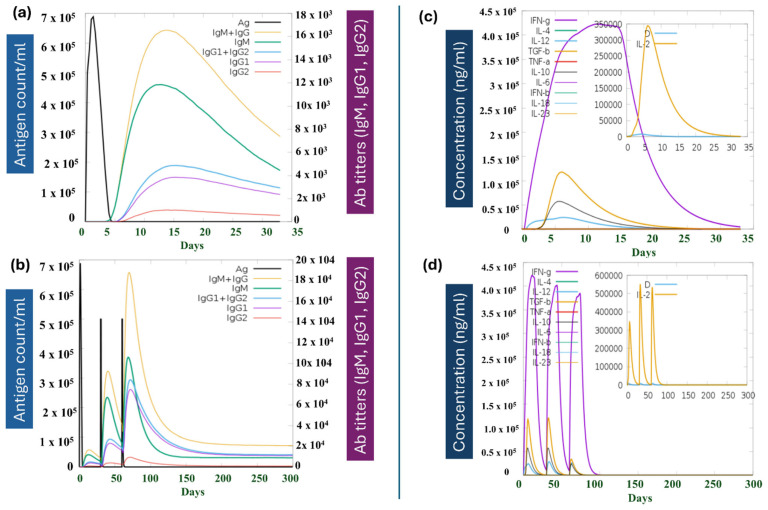
Illustration of the immune simulation findings. (**a**) The simulated immune response over time (in days) for first dose of the vaccine; the black curve shows the antigen count, which peaks rapidly around day 1 and then declines sharply as the immune response eliminates the antigen. (**b**) The cytokine and interleukin responses over time (in days) after first dose. (**c**) The effect of a final (second booster) dose at day 60. (**d**) The cytokine dynamics after the final (second booster dose) at day 60.

The prediction results after the first dose of MPXV-2-Beta showed that the antigen count peaked on day 1 at the level of 7 × 10^5^, followed by a rapid decrease in the count, which indicates the activation of the immune system. The initial IgM peak (11 × 10^3^) highlights the primary immune response. The later rise in IgG levels (especially IgG1 and IgG2) (3 × 10^3^) exhibited the induction of a comprehensive, robust and specific secondary IR. The high levels of IgG antibodies signify effective immunological memory formation, rendering rapid and efficient antigen clearance upon subsequent exposures (Figure 10a). Interferon-gamma (IFN-γ) exhibited the most pronounced peak around day 14 (4.3 × 10^5^ ng/mL). Interleukin-2 (IL-2) peaked initially on day 9 (29 × 10^4^ ng/mL) and was appropriate for facilitating T-cell proliferation and differentiation. Its rapid consequent decline indicates a well-regulated IR (Figure 10c). The cytokine simulation results demonstrate a coordinated IR, where early activators (like IL-2) initiate IR, and later IFN-γ sustained and enhanced immunological functions (Figure 10c). Additionally, the two booster doses at days 30 and 60 were administered to assess the capability of MPXV-2-Beta for inducing sustained primary and secondary IRs (Figure 10b). After the final (second) booster dose (Day 60), the antigen count (5 × 10^5^ count/mL) was observed; however, the peak was smaller compared with the peak developed after first exposure, suggesting that the immune system was well primed and speedily recognized and eliminated the antigen. Figure 10b represents the maximum total immunoglobulin response (IgM + IgG) achieved after the final booster dose was approximately (19 × 10^4^). Moreover, IgM appeared first, followed by a strong and prolonged IgG response, indicating the generation of immune memory (Figure 10b). The IgG subclasses (IgG1, IgG2) showed sustained levels, highlighting adaptive immunity development. Cytokine dynamic results highlighted the interplay of pro-inflammatory and regulatory cytokines, with IFN-γ (3.9 × 10^5^) and IL-2 (5.1 × 10^5^), which is significant for driving the immune response in a well-regulated fashion (Figure 10d). The simulation exhibited the booster dose’s effectiveness in consolidating and prolonging IRs. The IRs and their effect on the immunological cell population following the first booster doses are illustrated in Figure S1 of Supplementary File S2. Additionally, the effect of MPXV-2-Beta on the B-cell (B-lymphocytes) and T-cell (T-lymphocytes) populations is depicted in Figure S2 of Supplementary File S2.

### 3.11. Codon Optimization Followed by Gene Cloning of MPXV-2-Beta

**Figure 11:**
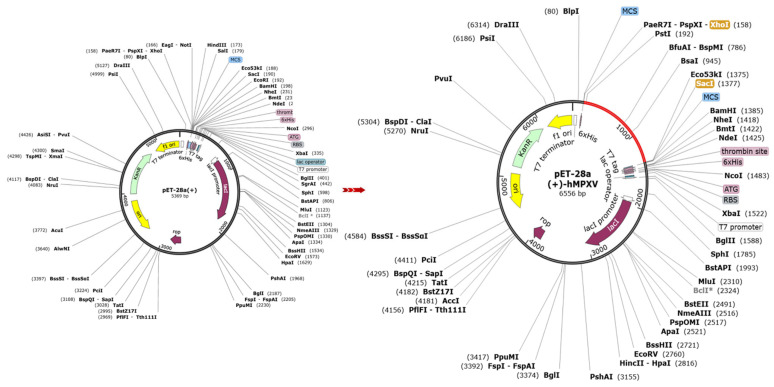
Molecular MPXV-2-Beta construct cloning in vector pET-28a (+).

Before protein expression evaluation, the codon optimization for efficient protein synthesis is important. The efficiency of MPXV-2-Beta construct expression is paramount for successful vaccine development. This research assessed the MPXV-2-Beta protein expression in an appropriate bacterial strain (*E. coli* K12) (Figure S1 of Supplementary File S3). The codon adaptation was executed for peak expression in the bacterial (*E. coli*) system. The adjusted and refined cDNA attained a CAI = 1.0 and the optimum value of the GC content (50.8599%) (Figure S1 of Supplementary File S3). Two (*n* = 02) restriction sites (EcoRI and XhoI) were deliberately incorporated into the MPXV-2-Beta construct’s N-terminal and C-terminal regions (Figure 11). Finally, cloning of the optimized proposed MPXV-2-Beta construct sequence was attained by adopting the appropriate expression vector (pET28a (+)) leveraging SnapGene, and a recombinant plasmid of length 6556 base pairs was obtained (Figure 11).

### 3.12. Determination of the Population Coverage for the MPXV-2-Beta Vaccine

**Figure 12:**
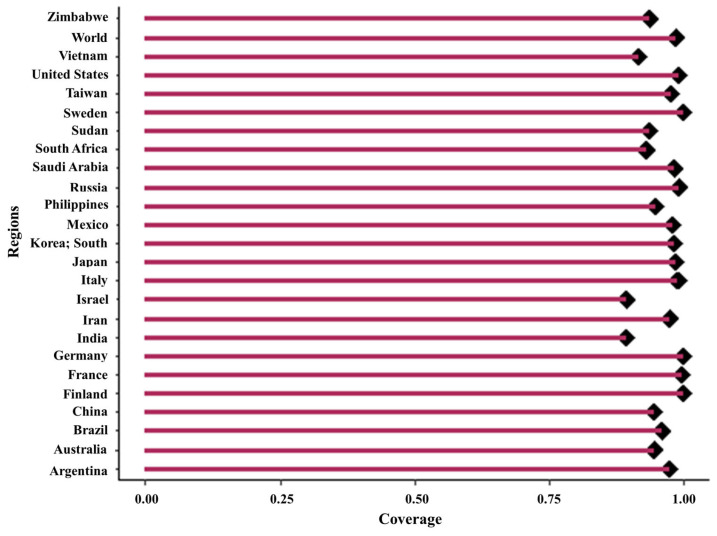
The population coverage of the MPXV-2-Beta construct across several countries.

The population coverage determination for the combined epitopes’ CTLEs and HTLEs revealed broad coverage worldwide. Notably, the selected epitopes achieved almost full world coverage (98.55%, SD = 10.41), emphasizing their effectiveness in targeting the diverse HLA alleles across different countries. This substantial population coverage exhibits the efficacy of the MPXV-2-Beta in providing immunity to hMPXV infection across diverse global populations (Figure 12).

## 4. Discussion

Preventing hMPXV outbreaks triggered by lineages/sub-lineages of hMPXV has posed greater challenges in recent years due to emerging human cases globally. Due to the lack of a definitive cure and vaccine for hMPXV, the currently in-use vaccines can only provide limited protection due to cross-reactivity with the vaccinia virus [76], necessitating novel strategies to develop vaccines and therapies to fight an outbreak of hMPXV. With no definitive treatment for hMPXV, first-line prophylactic intervention is only a preventive measure to curb the hMPXV outbreaks. Considering all of these relevant aspects, the current research aimed to design and simulate potential multi-epitope vaccine constructs and screen for the most potent vaccine constructs against hMPXV by targeting IMV proteins (A29L, E8L, H3L and M1R) which are crucial for viral pathogenesis.

Reverse vaccinology facilitates the identification of non-paralogous and conserved proteins from complete proteomes, eliminating redundancy to impart precise immune activation and minimizing the off-target effects [77]. The combination of bioinformatics, the availability of proteomic data, and reverse vaccinology has recently emerged as a potential technology with an edge over traditional methods to assist effective vaccine design [78]. The multi-epitope-based vaccines, which have the edge over single-epitope-based vaccines, offer a potent and viable approach, with limited adverse effects (i.e., is relatively safe), by which to accelerate vaccine development and prevent outbreaks of viral diseases. The unique properties of multi-epitope-based vaccines comprise the recognition of multiple HLA epitopes by TCRs, the capability of inducing both humoral and cell-mediated IRs (because it encompasses overlapping CTLEs, HTLEs and LBEs), broad range targeting of the microbial elements (because it is based on multiple antigenic target proteins), and flexibility to add appropriate adjuvants to boost IRs, which render them advantageous over single-epitope vaccines [79,80]. Moreover, the probability of pathological IRs and immune-modulating response (side effects) is minimized because they rarely contain undesirable microbial elements [81,82]. With efficacy limitations and safety concerns of existing live-attenuated vaccines like ACAM2000 and JYNNEOS, especially in immunocompromised or pediatric populations, and in the absence of definitive treatment for hMPXV infection [83,84], the proposed MPXV-2-Beta formulation could offer a safer and more effective vaccine, targeting the viral entry mechanism for combating hMPXV. These MPXV-2-Beta vaccines also offer flexibility in redesign to address emerging variants/clades of the hMPXV and offer safety by avoiding exposure to the virus entirely. The MPXV-2-Beta prototype has strain adaptability that could be helpful in fighting the evolving strains/clades of the hMPXV and curbing the zoonotic outbreak.

Identification and screening for the most suitable epitopes (LBEs, CTLEs and HTLEs) by targeting non-paralogous viral target proteins is paramount in vaccine development [85]. Moreover, a non-natural, pan-DR epitope with sequence AKVAAWTLKAAAC, known for activating CD4+ T cells, was added to the MPXV-2-Beta sequence to improve the vaccine’s potency and effectiveness [86]. The MITD is essential for directing CTLEs to the MHC 1 compartment to boost the antigen presentation [87]. Thus, the MITD sequence was also added to the construct. Allergenicity and toxicity to the host are critical factors for developing a safe vaccine; therefore, the epitopes included in vaccine constructs were first carefully screened to be nontoxic and nonallergenic. Subsequently, the vaccine constructs were reevaluated to ensure that they are nontoxic and nonallergenic and thus safe for downstream analysis [88].

Assessing the antigenicity of the epitopes and the vaccine constructs at an early phase of the development of the vaccine construct is significant; thus, each epitope incorporated into the final construct, followed by each vaccine construct, which were evaluated for the appropriate degree of antigenicity [88]. Adding adjuvants to the vaccine constructs boosts the IRs [89]. Thus, initially, two constructs were developed by combining LBEs, CTLEs, and HTLEs, which were subsequently linked to three different adjuvants to get six vaccine constructs. All six constructs were critically examined for their physiochemical properties, antigenicity, nontoxicity, nonallergenic and expression solubility, to screen out the most appropriate vaccine prototype (MPXV-2-Beta). In addition, a well-optimized 3D structure is vital for evaluating its molecular interactions with host immune receptor proteins. Structural integrity plays a significant role in ensuring proper antigen recognition and enhancing immune activation. Therefore, the secondary and 3D structure of MPXV-2-Beta was deduced and refined before the docking study [90]. ProSA (Z-score = −9.38) and Ramachandran plot (94.7% in favored regions plus 4.1% in allowed regions) for the 3D model of MPXV-2-Beta (molecular weight = 43 kDa) demonstrated that the construct was thermodynamically stable, structurally accurate and appropriately compatible for docking. Moreover, the MPXV-2-Beta showed high solubility, which is significant for functional studies [91]. Docking was executed to evaluate the interaction capability of the proposed MPXV-2-Beta with TLRs (TLR4 and TLR2) available on immune cells, which recognize pathogen-associated molecular patterns (PAMPs) and activate innate immune signaling to initiate adaptive IRs [92].

Additionally, NMA and MDS are necessary to evaluate biomolecular complexes’ stability, flexibility, and dynamic behavior under physiological conditions to ensure their functional reliability. After docking, the docked complexes NMA. The finding suggests that the TLR-4–MPXV-2-Beta complex is a stable conformation with a negative eigenvalue (0.3825626 × 10^−4^), which infers that the complex is allowed for significant collective motions, essential for functional flexibility (binding interactions or conformational changes). NMA and docking results indicate the strong and stable interaction TLR4 with MPXV-2-Beta, thereby implying the MPXV-2-Beta’s potential to elicit stable IR. Another TLR (TLR2) offers a protective role (by identifying viral components and triggering a pro-inflammatory response) in immune cell recruitment to clear pathogens [93]. The eigenvalues (0.50417 × 10^−5^) of TLR2–MPXV-2-Beta obtained from dynamic simulation suggest that the MPXV-2-Beta construct is tightly bound to the receptor, leading to a stable/rigid immune complex [94]. Moreover, the docking, followed by the NMA of MPXV-2-Beta with MHC (class I and class II) molecules, demonstrated a reliable interaction with low eigenvalues (0.1261 × 10^−4^ and 0.1647 × 10^−4^). The formation of a stable MPXV-2-Beta–MHC docked complex suggests that the complex could efficiently trigger adaptive IRs under physiological conditions [95]. The MDS confers an understanding of molecular complexes, such as TLR vaccines [96]. The vaccine alone and TLR vaccines exhibited RMSD values that stabilized within the first 20–30 ns of the 100 ns simulation, suggesting structural integrity, a critical factor for determining consistent biological activity [96]. RMSF analysis revealed that the majority of residues remained relatively rigid, with small fluctuations suggesting stable conformations over time [97]. The radius of gyration (to evaluate compactness) and hydrogen bond, a matrix or interaction, revealed compactness and strong intermolecular interactions between TLR–vaccine complexes, indicating vaccine–receptor binding, which was further validated by the MMPBSA ΔGbind observed for TLR2/TLR4 vaccines [98,99]. Gromac potential energy, short-range electrostatic energy, and kinetic energy also indicated the structural integrity, interTLR–vaccine electrostatic interactions, and thermal stability of the complexes [100]. Additionally, the stable simulation trajectory of total SAS area and SAS volume for TLR–vaccine complexes during 100 ns suggested the structural stability required for antigenic presentation [90,101]. Overall, NMA, MDS, post-MDS analyses exhibited the thermodynamically stable interaction between TLR and vaccine.

The *E. coli* K12 strain bacterial system is commonly used for large-scale recombinant protein production, necessitating codon optimization for achieving efficient expression [102]. A CAI = 1.0 and GC content (50.8599%) was achieved, which ensures desirable protein yield. Optimal expression necessitates CAI > 0.890 with GC content (30–70%) [103].

**Figure 13:**
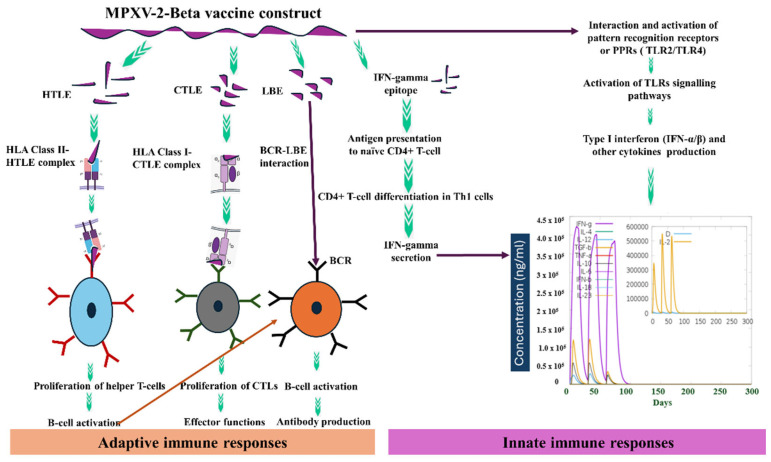
Schematics of the proposed MXPV-2-Beta construct’s probable mechanism of action.

Because the final construct comprises CTL, HTL, IFN-γ epitopes, and LBE, our hope is that it triggers the activation and proliferation of the respective immune cells in the host, which could further result in the activation of other immune cells through a complex signaling mechanism. Antigen clearance, the initial production of a substantial titer of IgM, followed by a rise in the titer of the IgG during immunological simulation, signifies the strong interaction of the MHC classes I and II with the proposed construct [89]. Furthermore, cytokine dynamic simulation results show the production of pro-inflammatory and regulatory cytokines, which is significant for driving the immune response in a well-regulated fashion (Figure 10d), highlighting the strong interaction of the MPXV-2-Beta constructs with pathogen recognition receptors (PPRs) such as TLR2 and TLR4 [104]. A schematic has been developed to demonstrate the probable mechanism of action of the MPXV-2-Beta construct in eliciting both innate and adaptive immune responses (Figure 13).

Achieving broad population coverage is essential for vaccine efficacy across diverse demographics. This study evaluated the vaccine’s potential to induce strong immune responses (IRs) in a global population. Population coverage was estimated by analyzing the binding affinity of each antigenic determinant (epitope) to its corresponding HLA.

## 5. Limitations

The current analysis proposes a multi-epitope vaccine (MPXV-2-Beta) targeting the hMPXV protein, though limitations remain. In the current research only IMV-antigenic proteins were targeted for the prediction of antigenic peptides to construct the vaccine but incorporating the EEV-derived epitopes with IMV-derived epitopes in vaccine construct could further enhance the efficacy of the vaccine constructs, which in turn underscores one of the limitations of this study. Moreover, immunoinformatics-based design relies on predictive models, making protection levels uncertain. Challenges include a lack of benchmarking, limited predictive methods, and insufficient datasets. Despite recent vaccine successes, the MPXV-2-Beta construct requires in vitro and in vivo bioassays for safety and efficacy validation.

## 6. Conclusions

This study utilized the reverse vaccinology approach and immunoinformatics method to design and propose a novel multi-epitope vaccine against hMPXV, a well-recognized emerging threat to public health. Initially, six constructs were devised. One of the constructs was screened as the most appropriate prototype for further analysis based on physiochemical properties and antigenicity. Docking, followed by dynamic simulation assessment of MPXV-2-Beta with TLR and MHC molecules, demonstrated stable MPXV-2-Beta immune receptors (TLRs and MHCs) for recognition, immune activation and recognition and generation of sustained and robust IRs. MPXV-2-Beta was the optimal construct, exhibiting strong immunogenicity, TLR binding affinity, and stable gene expression in *E. coli*. The combined world population coverage of the MPXV-2-Beta was high (98.55%, SD = 10.41). Though the multi-epitope vaccine presents a promising first-line prophylactic approach for preventing hMPXV outbreaks, Additional validation through experimental and clinical studies is sought to verify its safety and efficacy.

## Figures and Tables

**Table 1:** Baseline physical and chemical attributes of the target hMPXV/IMV proteins’ representative sequences for vaccine design.

Proteomes	M1R/IMV	E8L/IMV	H3L/IMV	A29L/IMV
Allergenicity status	NA	NA	NA	NA
Antigenicity/(score)	Ag/(0.6339)	Ag/(0.5316)	Ag/(0.4538)	N-Ag/(0.3277)
Residue toxicity	Nontoxic	Nontoxic	Nontoxic	Nontoxic
AA Length	250	304	324	110
Mw/TpI	27,303.24/6.72	35,247.99/7.77	37,531.34/5.53	12,559.24/5.73
AI	87.48	88.22	97.99	73.73
GRAVY	−0.004	−0.359	−0.02	−0.75
I-i	33.47	45.45	41.77	32.28
EHL-MR	30 h	30 h	30 h	30 h
EHL-Y	>20 min	>20 min	>20 min	>20 min
EHL-E	>10 h	>10 h	>10 h	>10 h
EC (M^−1^cm^−1^)	19,285	56,270	38,390	2980
E. solubility	0.853	0.847	0.882	0.74

IMV = intracellular mature virus, NA = non-allergen, AA = amino acid, N-Ag = non-antigenic, Ag = antigenic, Mw = molecular weight, AI = aliphatic index, Tpi = theoretical pI, pI = isoelectric point, GRAVY = grand average of hydropathicity, Ii = instability index EHL-MR = estimated half-life in mammalian reticulocytes or invitro, EHL-Y = estimated half-life in yeast or in vivo, EHL-E = estimated half-life in *E. coli* or in vivo, E. solubility = expression solubility, and EC = extinction coefficient measured in water at 280.

**Table 2:** The immunogenic potential of the prioritized LBEs and promiscuous CTLEs.

Proteins IDs/Proteins	LBL-Epitope Designation	LBL-Epitope	ABCP(s)	Ag(s)	Toxicity	Allergenicity
QJQ40281.1/A29L/IMV	LBE-1	LRAAMISLAKKIDVQT	0.71	0.7326	Nontoxic	NA
	LBE-2	TLKQRLTNLEKKITNI	0.69	0.9498	Nontoxic	NA
QJQ40248.1/E8L/IMV	LBE-3	ARLKTLDIHYNESKPT	0.74	1.0282	Nontoxic	NA
	LBE-4	LVRINFKGGYISGGFL	0.68	1.1759	Nontoxic	NA
AGR38652.1/H3L/IMV	LBE-5	NDDPDHYKDYVFIQWT	0.87	0.9084	Nontoxic	NA
	LBE-6	PNFWSRIGTVAAKRYP	0.78	1.0417	Nontoxic	NA
QJQ40223.1/M1R/IMV	LBE-7	IEIGNFYIRQNHGCNI	0.89	1.148	Nontoxic	NA
	LBE-8	DECYGAPGSPTNLEFI	0.88	0.88	Nontoxic	NA
**Proteins IDs/Proteins**	**CTL-Epitope** **Designation**	**CTL-Epitope**	**IC50-value**	**Ag(s)**	**Toxicity**	**Allergenicity**
QJQ40281.1/A29L/IMV	CTL-1	TLRAAMISL	51.12	1.1323	Nontoxic	NA
	CTL-2	TLKQRLTNL	12.59	0.9942	Nontoxic	NA
QJQ40248.1/E8L/IMV	CTL-3	RLKTLDIHY	34.19	1.9035	Nontoxic	NA
	CTL-4	SDLREACFSY	48.1	1.7231	Non-toxic	NA
AGR38652.1/H3L/IMV	CTL-5	RIGTVAAKR	50.9	1.7688	Nontoxic	NA
	CTL-6	RIGTVAAKRY	25.62	1.44	Nontoxic	NA
QJQ40223.1/M1R/IMV	CTL-7	LANKENVHW	5.4	1.716	Nontoxic	NA
	CTL-8	LTPEQKAYV	31.4	1.1239	Nontoxic	NA

IMV = intracellular mature virus, LBE = linear B-cell epitope, ABCP(s) = ABCPred-score, Ag(s) = antigenicity score (Vaxijen score), and NA = non-allergen.

**Table 3:** The immunogenic potential of the prioritized promiscuous MHC-class-2 binders (IL-4 inducer overlapped) and IFN-gamma epitopes (IL-4 inducer overlapped).

Proteins IDs/Proteins	HTL-Epitope Designation	HTL-Epitope	IC50-Value	Ag(s)	II/IL-4 Score	Tox/Aller
QJQ40281.1/A29L/IMV	HTL -1	TLRAAMISLAKKIDV	23.9	0.8881	II/0.25	NT/NA
	HTL -2	NLEKKITNITTKFEQ	83.2	0.6727	II/0.31	NT/NA
QJQ40248.1/E8L/IMV	HTL -3	LDYFTYLGTTINHSA	8.8	0.6462	II/1.05	NT/NA
	HTL -4	TLDIHYNESKPTTIQ	20.4	0.7176	II/1.35	NT/NA
AGR38652.1/H3L/IMV	HTL -5	LQMREIITGNKVKTE	65.6	0.834	II/0.26	NT/NA
	HTL -6	PDHYKDYVFIQWTGG	74.4	0.649	II/0.32	NT/NA
QJQ40223.1/M1R/IMV	HTL -7	PAMFTAALNIQTSVN	6.5	0.4876	II/0.34	NT/NA
	HTL -8	NDKIKLILANKENVH	15.1	0.56	II/1.45	NT/NA
**Proteins IDs/Proteins**	**IFN-γ-Epitope Designation**	**IFN-Gamma Epitope**	**IC50-Value**	**Ag(s)**	**IFN-γ/IL-4 Scores**	**Tox/Aller**
QJQ40281.1/A29L/IMV	IFN-γ -1	KQRLTNLEKKITNIT	74.4	0.986	0.9657/0.31	NT/NA
	IFN-γ -2	LEKKITNITTKFEQI	100.4	0.753	0.6529/0.31	NT/NA

IMV = intracellular mature virus, IFN-γ = interferon gamma, HTL = helper T-cell, Ag(s) = antigenicity score (Vaxijen), IL-4 = interleukin-r, II = IL-4 inducing, Tox = toxicity, Aller = allergenicity, NT = non-toxic, and NA = non-allergenic.

**Table 4:** Antigenicity, allergenicity status, toxigenic profile and physiochemical characteristics of the six target models of the vaccine construct.

Parameters	MPX-1- Beta	MPXV-1- Hbha	MPXV-1- Ribos	MPXV-2- Beta	MPXV-2-Hbha	MPXV-2-Ribos
Allergenicity	NA	NA	NA	NA	NA	NA
Antigenicity/score	Ag/0.7495	Ag/0.6912	Ag/0.6752	Ag/0.7202	Ag/0.6686	Ag/0.6513
Residue toxicity/score	Nontoxic/0.29	Nontoxic/0.24	Nontoxic/0.24	Nontoxic/0.52	Nontoxic/0.26	Nontoxic/0.24
AA Length	405	519	490	407	521	492
Mw/TpI	42,760.94/8.7	55,228.39/9.17	51,040.22/9.17	43,102.1/9.77	55,569.55/9.2	51,381.39/9.2
AI	71.53	77.9	80.49	65.63	73.25	75.57
GRAVY	−0.465	−0.455	−0.273	−0.597	−0.557	−0.382
I-i	26.42	30.20	23.08	25.92	29.79	22.68
EHL-MR	1 h	1 h	1 h	1 h	1 h	1 h
EHL-Y	30 min	30 min	30 min	30 min	30 min	30 min
EHL-E	>10 h	>10 h	>10 h	>10 h	>10 h	>10 h
EC (M^−1^cm^−1^)	47,705	48,820	44,350	50,810	51,925	47,330
E. solubility	1.107	0.884	0.839	1.167	0.932	0.921
E. solubility/Soluproscore	SE/0.836	ISE/0.346	SE/0.498	SE/0.942	SE/0.605	SE/0.624

IMV = intracellular enveloped virus, NA = non-allergen, AA = amino acid, N-Ag = non-antigenic, Ag = antigenic, Mw = molecular weight, AI = aliphatic index, GRAVY = grand average of hydropathicity, Tpi = theoretical pI, pI = isoelectric point, Ii = instability index, EHL-MR = estimated half-life (in mammalian reticulocytes or invitro), EHL-Y = estimated half-life in yeast or in vivo, EHL-E = estimated half-life in *E. coli* or in vivo, E. solubility = expression solubility, SE = soluble expression, ISE = insoluble expression, Soluproscore cut off > 0.5, and EC = extinction coefficient measured in water at 280.

**Table 5:** Quantitative details of the interacting residues, polar contacts, and interface areas in TLR–MPXV-2-Beta and MHC–MPXV-2-Beta docked complexes.

Complex Description	No. of Residues in the Interface	Count of Salt Bridges	Count of Hydrogen Bonds	Count of Non-Bonded Contacts	Interface Area (Å^2^)
TLR4	41	04	28	206	1709
MPXV-2-Beta	31				1936
TLR2	23	05	13	159	1112
MPXV-2-Beta	27				1030
MHC-I–MPXV-2-Beta complex	12–13 (A–C)	09 (A–C)	09 (A–C)	102 (A–C)	671–663 (A–C)
	20–22 (B–C)	6 (B–C)	10 (A–C)	146 (A–C)	1071–1081 (B–C)
MHC-II–MPXV-2-Beta complex	14–16 (A–C)	03 (A–C)	9 (A–C)	107 (A–C)	688–675 (A–C)
	4–4 (B–C)	4 (B–C)	4 (B–C)	30 (B–C)	188–170 (B–C)

A = chain A of MHC class-I and MHC class-II. C = chain of proposed MPXV-2-Beta vaccine.

## Data Availability

All original data and contributions from this study are available in the article and Supplementary Materials. For further inquiries, corresponding authors can be contacted.

## Supplementary Materials

The following supporting information can be downloaded at: https://www.mdpi.com/article/10.3390/biomedicines13061439/s1.
