# Supplementary material for: Design and Validation of a Multi-Epitope mRNA Vaccine Construct Against Human Monkeypox Virus (hMPXV) by Annotating Protein of Intracellular Mature Virus (IMV) Form of hMPXV
Source: Biomedicines. 2025 Jun 11;13(6):1439. doi: 10.3390/biomedicines13061439 (PMC12190101; doi:10.3390/biomedicines13061439)
Supplement: Supplementary file 1 [file biomedicines-13-01439-s001.zip › Supplementary File S1.pdf]

```
>Cluster 4
0       304aa, >QJQ40248.1... *
1       304aa, >AGR38664.1... at 99.34%
2       304aa, >AGR38473.1... at 99.67%
3       304aa, >AGR38282.1... at 99.67%
4       304aa, >AGR38091.1... at 99.67%
5       304aa, >AGR37901.1... at 99.34%
6       304aa, >AGR37711.1... at 99.34%
7       304aa, >AGR37521.1... at 99.34%
8       304aa, >AGR37330.1... at 99.67%
9       304aa, >AGR37139.1... at 99.67%
10      304aa, >AGR36948.1... at 99.67%
11      304aa, >AGR36757.1... at 99.67%
12      304aa, >AGR36566.1... at 99.67%
13      304aa, >AGR36375.1... at 99.67%
14      304aa, >AGR36184.1... at 99.67%
15      304aa, >AGR35993.1... at 99.67%
16      304aa, >AGR35802.1... at 99.67%
17      304aa, >AGR35611.1... at 99.67%
18      304aa, >AGR35420.1... at 99.67%
19      304aa, >AGR35229.1... at 99.67%
20      304aa, >AGR35038.1... at 99.67%
21      304aa, >AGR34847.1... at 99.67%
[illegible]
```

CD-Hit cluster QJQ40248.1/E8L/IMV

```
>Cluster 7
0       110aa, >QJQ40281.1... *
1       110aa, >AGR38698.1... at 98.18%
2       110aa, >AGR38507.1... at 98.18%
3       110aa, >AGR38316.1... at 98.18%
4       110aa, >AGR38125.1... at 98.18%
5       110aa, >AGR37935.1... at 98.18%
6       110aa, >AGR37745.1... at 98.18%
7       110aa, >AGR37555.1... at 98.18%
8       110aa, >AGR37364.1... at 98.18%
9       110aa, >AGR37173.1... at 98.18%
10      110aa, >AGR36982.1... at 98.18%
11      110aa, >AGR36791.1... at 98.18%
12      110aa, >AGR36600.1... at 98.18%
13      110aa, >AGR36409.1... at 98.18%
14      110aa, >AGR36218.1... at 98.18%
15      110aa, >AGR36027.1... at 98.18%
16      110aa, >AGR35836.1... at 98.18%
17      110aa, >AGR35645.1... at 98.18%
18      110aa, >AGR35454.1... at 98.18%
19      110aa, >AGR35263.1... at 98.18%
20      110aa, >AGR35072.1... at 98.18%
21      110aa, >AGR34881.1... at 98.18%
```

CD-Hit cluster=QJQ40281.1/A29L/IMV

```
>Cluster 7
0       250aa, >QJQ40223.1... *
1       250aa, >AGR38639.1... at 100.00%
2       250aa, >AGR38448.1... at 100.00%
3       250aa, >AGR38257.1... at 100.00%
4       250aa, >AGR38066.1... at 100.00%
5       250aa, >AGR37876.1... at 100.00%
6       250aa, >AGR37686.1... at 100.00%
7       250aa, >AGR37496.1... at 100.00%
8       250aa, >AGR37305.1... at 100.00%
9       250aa, >AGR37114.1... at 100.00%
10      250aa, >AGR36923.1... at 100.00%
11      250aa, >AGR36732.1... at 100.00%
12      250aa, >AGR36541.1... at 100.00%
13      250aa, >AGR36350.1... at 100.00%
14      250aa, >AGR36159.1... at 100.00%
15      250aa, >AGR35968.1... at 100.00%
16      250aa, >AGR35777.1... at 100.00%
17      250aa, >AGR35586.1... at 100.00%
18      250aa, >AGR35395.1... at 100.00%
19      250aa, >AGR35204.1... at 100.00%
20      250aa, >AGR35013.1... at 100.00%
[illegible]
```

CD-Hit cluster QJQ40223.1/M1R/IMV

```
>Cluster 1
0       324aa, >AGR38652.1... *
1       324aa, >AGR38461.1... at 100.00%
2       324aa, >AGR38270.1... at 100.00%
3       324aa, >AGR38079.1... at 100.00%
4       324aa, >AGR37889.1... at 100.00%
5       324aa, >AGR37699.1... at 100.00%
6       324aa, >AGR37509.1... at 100.00%
7       324aa, >AGR37318.1... at 100.00%
8       324aa, >AGR37127.1... at 100.00%
9       324aa, >AGR36936.1... at 100.00%
10      324aa, >AGR36745.1... at 100.00%
11      324aa, >AGR36554.1... at 100.00%
12      324aa, >AGR36363.1... at 100.00%
13      324aa, >AGR36172.1... at 100.00%
14      324aa, >AGR35981.1... at 100.00%
15      324aa, >AGR35790.1... at 100.00%
16      324aa, >AGR35599.1... at 100.00%
17      324aa, >AGR35408.1... at 100.00%
18      324aa, >AGR35217.1... at 100.00%
19      324aa, >AGR35026.1... at 100.00%
20      324aa, >AGR34835.1... at 100.00%
21      324aa, >AGR34644.1... at 100.00%
```

CD-Hit cluster AGR38652.1/H3L/IMV

```
>QJQ40281.1 A29L [Monkeypox virus]
MDGTLFPGDDDLAIPATEFFSTKAAKNPETKREAIVKAYGDDNEETLKQRLTNLEKKITNITTKFEQIEK
CCKRNDEVLFRLENHAETLRAAMISLAKKIDVQTGRHPYE

>QJQ40248.1 E8L [Monkeypox virus]
MPQQLSPINIETKKAISDARLKTLDIHYNESKPTTIQNTGKLVRINFKGGYISGGFLPNEYVLSTIHIYW
GKEDDYGSNHLIDVYKYSGEINLVHWNKKKYSSYEEAKKHDDGIIIIAIFLQVSDHKNVYFQKIVNQLDS
IRSANMSAPFDSVFYLDNLLPSTLDYFTYLGTTINHSADAAWIIFPTPINIHSDQLSKFRTLLSSSNHEG
KPHYITENYRNPYKLNDDTQVYYSGEIIRAATTSPVRENYFMKWLSDLREACFSYYQKYIEGNKTFAIIA
IVFVFILTAILFLMSQRYSREKQN

>AGR38652.1 H3L [Monkeypox virus]
MAAAKTPVIVVPVIDRPPSETFPNVHEHINDQKFDDVKDNEVMQEKRDVVIVNDDPDHYKDYVFIQWTGG
NIRDDDKYTHFFSGFCNTMCTEETKRNIARHLALWDSKFFIELENKNVEYVVIIENDNVIEDITFLRPVL
KAIHDKKIDILQMREIITGNKVKTELVIDKDHAIFTYTGGYDVSLSAYIIRVTTALNIVDEIIKSGGLSS
GFYFEIARIENEMKINRQIMDNSAKYVEHDPRLVAEHRFETMKPNFWSRIGTVAAKRYPGVMYTFTTPLI
SFFGLFDINVIGLIVILFIMFMLIFNVKSKLLWFLTGTFVTAFI

>QJQ40223.1 M1R [Monkeypox virus]
MGAAASIQTTVNTLSERISSKLEQEANASAQTKCDIEIGNFYIRQNHGCNITVKNMCSADADAQLDAVLS
AATETYSGLTPEQKAYVPAMFTAALNIQTSVNTVVRDFENYVKQTCNSSAVVDNKLKIQNVIIDECYGAP
GSPTNLEFINTGSSKGNCAIKALMQLTTKATTQIAPRQVAGTGVQFYMIVIGVIILAALFMYYAKRMLFT
STNDKIKLILANKENVHWTTYMDTFFRTSPMIIATTDIQN
```

Figure S1. CD-Hit clusters and sequences of the target protein with their accession numbers.

SOPMA result for : UNK_12496330

Abstract Geourjon, C. & Deléage, G., SOPMA: Significant improvement in protein secondary s

```
        10        20        30        40        50        60        70
         |         |         |         |         |         |         |
EAAAKGIINTLQKYYCRVRGGRCAVLSCLPKEEQIGKCSTRGRKCCRRKKEAAAKAKFVAAWTLKAAAGG
hhhhhhhhhhhhhhhhhhhhhhhhhhhhhhhhhhhhhhhhhhhhhhhhhhhhhhhhhhhhhhhhhhhhhh
GSTLKQRLTNLEKKITNIGPGPGKKNLEKKITNITTKFEQAAYTLKQRLTNLGPGPGKKKQRLTNLEKKI
hhhhhhhhhhhhhhhhhhhhhhhhhhhhhhhhhhhhhhhhhhhhhhhhhhhhhhhhhhhhhhhhhhhhhh
TNITGGGSHEYGAEALERAGGGGSLVRINFKGGYISGGFLGPGPGKKTLDIHYNESKPTTIQAAYSDLRE
hhhhhhhhhhhhhhhhhhhhhhhheeeeecccceeeccccccccccceeeeeecccccchhhhhhhhhhh
ACFSYGPGPGKKLEKKITNITTKFEQIGGGSHEYGAEALERAGGGGSPNFWSRIGTVAAKRYPGPGPGKK
heeecccccccchhhhhhhhhheeeeeccccchhhhhhhhhcccccccceehhhceeeeccccccccccc
PDHYKDYVFIQWTGGAAYRIGTVAAKRYGGGSHEYGAEALERAGGGGSDECYGAPGSPTNLEFIGPGPGK
ccccceeeeeeeccccdeeeeeeeeeecccccccchhhhhhhhhhccccccceecccccccceeeeecccccc
KNDKIKLILANKENVHAAYLTPEQKAYVHEYGAEALERAGAKFVAAWTLKAAAGGGS
cccceeeeeecccccheeeeccchhhhhhhhhhhhhhhhhhhhhhhhhhhhhccccc
```

Sequence length : 407

SOPMA :

Alpha helix	(Hh)	:	238 is	58.48%
3_{10} helix	(Gg)	:	0 is	0.00%
Pi helix	(Ii)	:	0 is	0.00%
Beta bridge	(Bb)	:	0 is	0.00%
Extended strand	(Ee)	:	61 is	14.99%
Beta turn	(Tt)	:	0 is	0.00%
Bend region	(Ss)	:	0 is	0.00%
Random coil	(Cc)	:	108 is	26.54%
Ambiguous states	(?)	:	0 is	0.00%
Other states		:	0 is	0.00%

Figure S2. Secondary structure elements of the MPXV-2-Beta.

Results for minimum free energy prediction

The optimal secondary structure in dot-bracket notation with a minimum free energy of **-454.40** kcal/mol is given below.

[color by base-pairing probability | color by positional entropy | no coloring]

```
1     CUUCGCCGCCGCUUUCCGUAAUAAUUGUGGGACGUCUUUAUAAUAACGGCGCACGCGCCGCCGGCGACGCGCCACGACUCGACGGACGGCUUUCUUCUUGUCUAACCGUUUACGUCGUGGGCGCCGGCGUUUACGACGGCGGUUUUUCUUCGCCGCC
160   GCUUUCGCUUUAAACACCGCCGCACCUGGGACUUUCGCCGCCGCCCGCCGCCGUCGUGGGACUUUGUCGCGGACUGGUUGGACCUUUUUUUUUAAUGGUUGUAACCGGGCCCGGGCCCGUUUUUUUUGGACCUUUUUUUUUAAUGGUUGUAAUGGUGGUU
320   UAAACUUGUCCGCCGCAUAUGGGACUUUGUCGCGGACUGGUUGGACCCGGGCCCGGGCCCGUUUUUUUUUGUCGCGGACUGGUUGGACCUUUUUUUUUAAUGGUUGUAAUGGCCGCCGCCGUCGGUACUUAUACCGCGCCUUCGCGACCUUGCGCGCCCG
480   CCGCCGCCGUCGGACCACGCGUAAUUGAAAUUUCCGCCGAUAUAAUCGCCGCCGAAAGACCCGGGCCCGGGCCCGUUUUUUUGGGACCUAUAAGUAAUAUUGCUUUCGUUUGGCUGGUGGUAAGUCCGCCGCAUAUCGCUAGACGCGCUUCGCACGAAAU
640   CGAUACCGGGCCCGGGCCCGUUUUUUGACCUUUUUUUUUAAUGGUUGUAAUGGUGGUUUAAACUUGUCUAACCGCCGCCGUCGGUACUUAUACCGCGCCUUCGCGACCUUGCGCGCCCGCCGCCGCCGUCGGGCUUGAAAACCUCGGCGUAACCGUGGCA
800   CCGCCGCUUUGCGAUAGGCCCGGGCCCGGGCCCGUUUUUUGGCCUAGUAAUAUUUCUAAUACACAAAUAAGUCACCUGGCCGCCGCGCCGCAUAGCGUAACCGUGGCACCGCCGCUUUGCGAUACCGCCGCCGUCGGUACUUAUACCGCGCCUUCGCGAC
960   CUUGCGCGCCCGCCGCCGCCGUCGCUACUUACGAUACCGCGCGGCCCGUCGGGCUGGUUGGACCUUAAAUAACCGGGCCCGGGCCCGUUUUUUUUGCUAUUUAAUUUGACUAAGACCGCUUGUUUCUUUUGCACGUACGCCGCAUAGACUGGGGCCUUG
1120  UCUUUCGCAUACACGUACUUAUACCGCGCCUUCGCGACCUUGCGCGCCCGCGCUUUAAACACCGCCGCACCUGGGACUUUCGCCGCCGCCCGCCGCCGUCG

1     ........((((...............))))...............((((((...(((.((.((((((....(((((((..(((((..(((.........)))...)))))...)))))))(((.(((.((((((((((((((((.........))))))
160   )...)))...))))).))).)))....(((.((((((.((.(((((((.......)))))....((((((((..((((((((.((......)).)))).)))))(((((((((((((.........((((.((......)).))))........((((
320   (((.((.((((((.(((..........))).)))))).)))))))))))))))))))))..........(((((((..(((..((((.((......)).))))...(((((....)))))(((((....))))).))))))))))).((((((.(((
480   .((....)).)))....)))))))........)))).)))))....)).)).)))))).)))(((((((((((((.((.((((((...(((.((.((((((((((..((((((((((((((.....))))))......))))))))......((.(((((.
640   ......(((((((((((((......((((.((......)).))))......(((((((...........)))))))......(((((....))))).((((((((((....(((.((..((((((((((..(((.........)))))))).....))))).
800   ..))))).))))))).)))).)))))))))))))..)))))))..)))))))))).)).)))......((((((.(((.(((.((.(((.((((.((((.....(((....)))))))).))))....))).)).))))))))))))..((((....)))).(
960   (..((..(((((.((..((((.(((..............))))))).)).)))))..)).))..)))))).)).)))))))))))))..........((..(((((.......)))))..))..(((((...(((..(((((..((..((((.........)
1120  )))...)).....)))))........((((...((((......))))...))))..............)))...)))))..)))))).))..))))))))))..
```

You can download the minimum free energy (MFE) structure in [Vienna Format| Ct Format]. You can get thermodynamic details on this structure by submitting to our RNAeval web server.

Results for thermodynamic ensemble prediction

The free energy of the thermodynamic ensemble is **-479.13** kcal/mol.

The frequency of the MFE structure in the ensemble is **0.00** %.

The ensemble diversity is **305.27**.

You may look at the **dot plot** containing the base pair probabilities [EPS|PDF|IMAGE CONVERTER].

The centroid secondary structure in dot-bracket notation with a minimum free energy of **-333.20** kcal/mol is given below.

[color by base-pairing probability | color by positional entropy | no coloring]

Figure S3. mRNA secondary structure of the MPXV-2-Beta and minimum free energies.
