# Supplementary material for: Design and Validation of a Multi-Epitope mRNA Vaccine Construct Against Human Monkeypox Virus (hMPXV) by Annotating Protein of Intracellular Mature Virus (IMV) Form of hMPXV
Source: Biomedicines. 2025 Jun 11;13(6):1439. doi: 10.3390/biomedicines13061439 (PMC12190101; doi:10.3390/biomedicines13061439)
Supplement: Supplementary file 1 [file biomedicines-13-01439-s001.zip › Supplementary File S2.pdf]

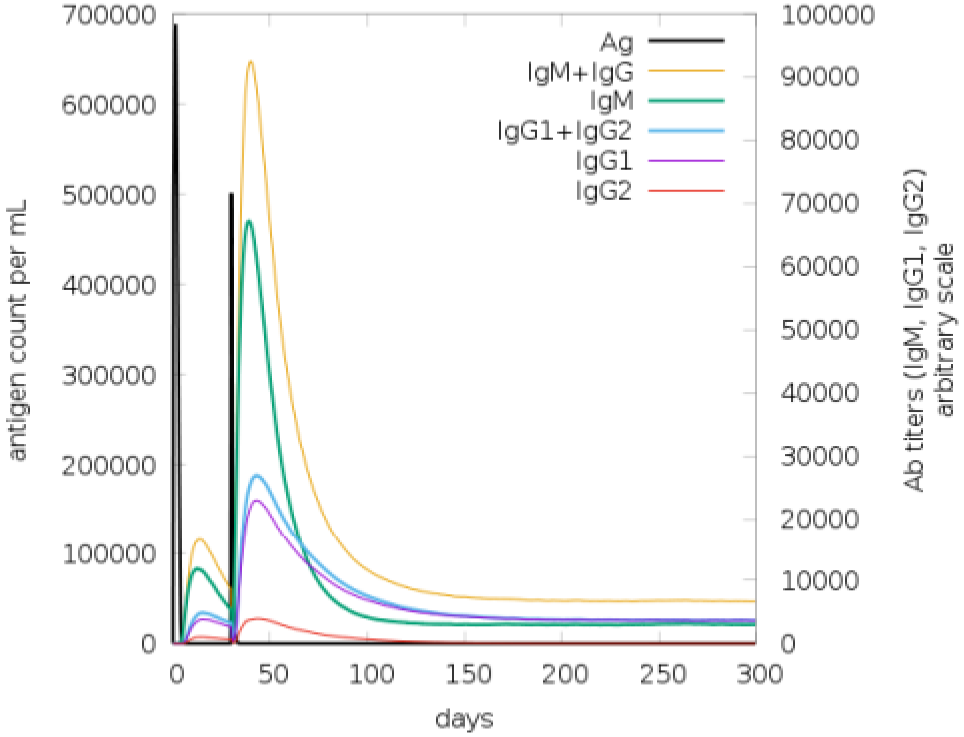

Antigen count and Ab titter

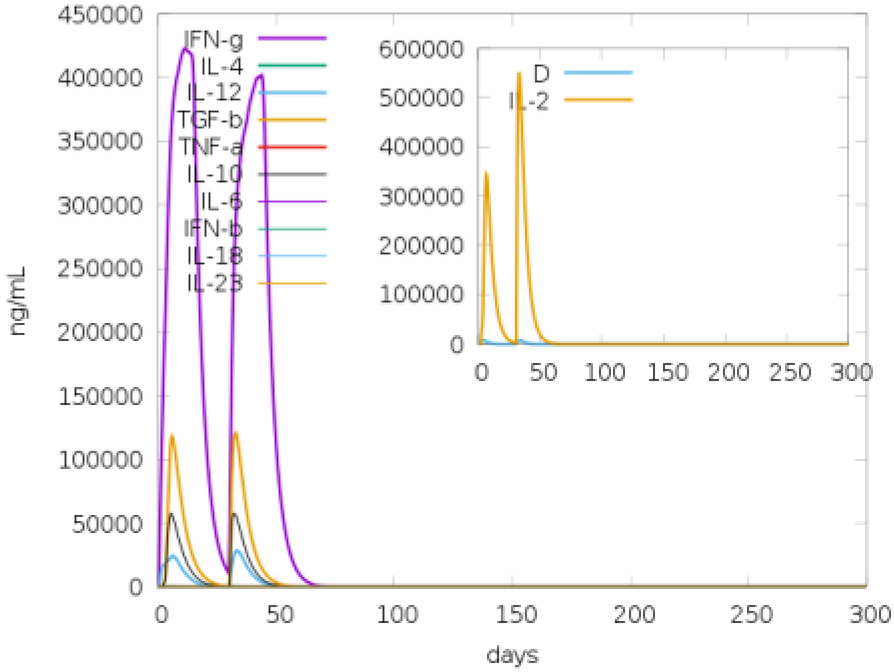

Cytokine dynamics

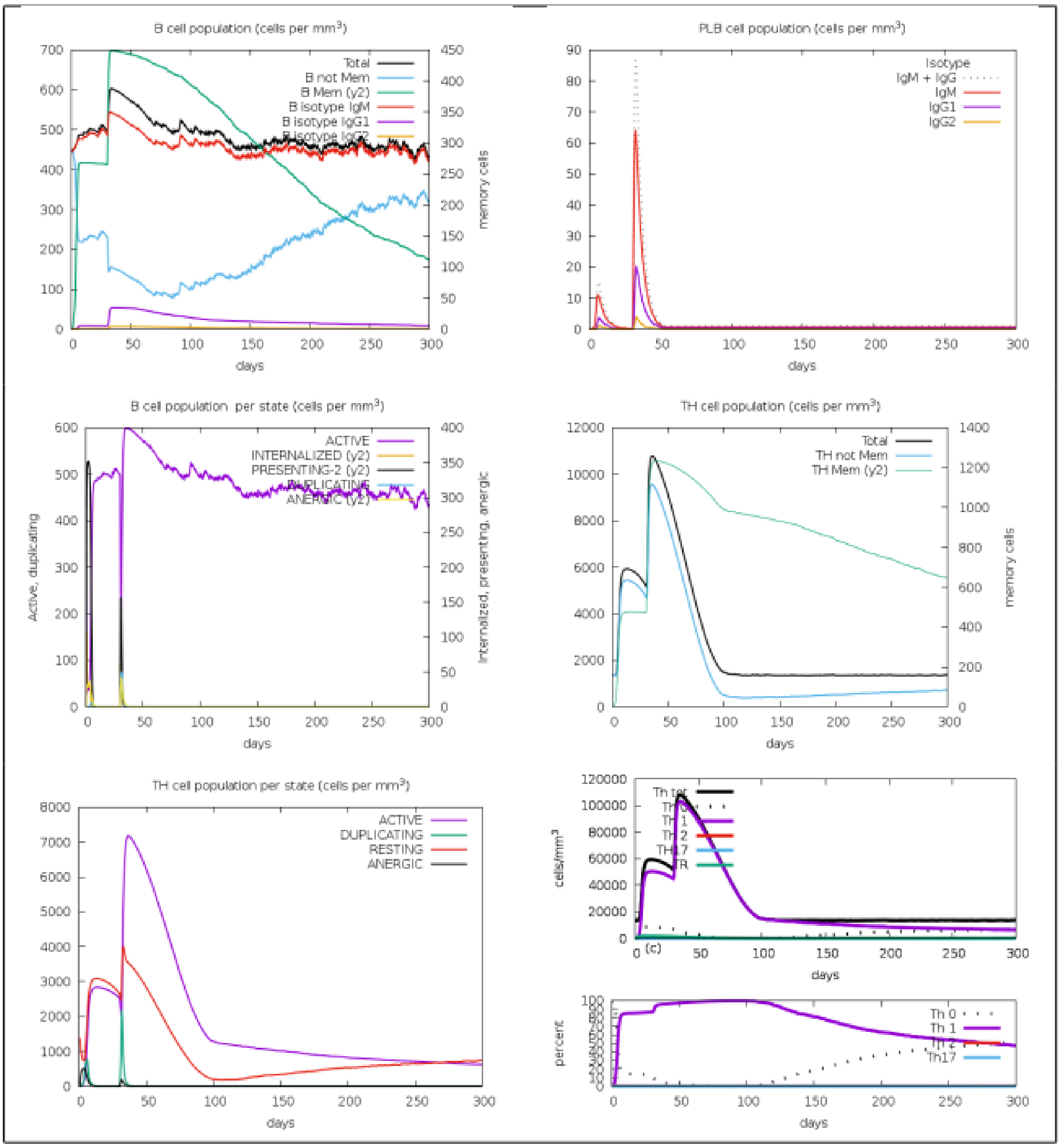
B cell population (cells per mm3)
Total
B not Mem
B Mem (y2)
B isotype IgM
B isotype IgG1
B isotype IgG2
memory cells
days
PLB cell population (cells per mm3)
Isotype
IgM + IgG
IgM
IgG1
IgG2
days
B cell population per state (cells per mm3)
ACTIVE
INTERNALIZED (y2)
PRESENTING-2 (y2)
DUPLICATING
ANERGIC (y2)
Active, duplicating
Internalized, presenting, anergic
days
TH cell population (cells per mm3)
Total
TH not Mem
TH Mem (y2)
memory cells
days
TH cell population per state (cells per mm3)
ACTIVE
DUPLICATING
RESTING
ANERGIC
days
Th 1
Th 2
cells/mm3
(c)
days
Th 0
Th 1
Th 2
Th17
percent
days

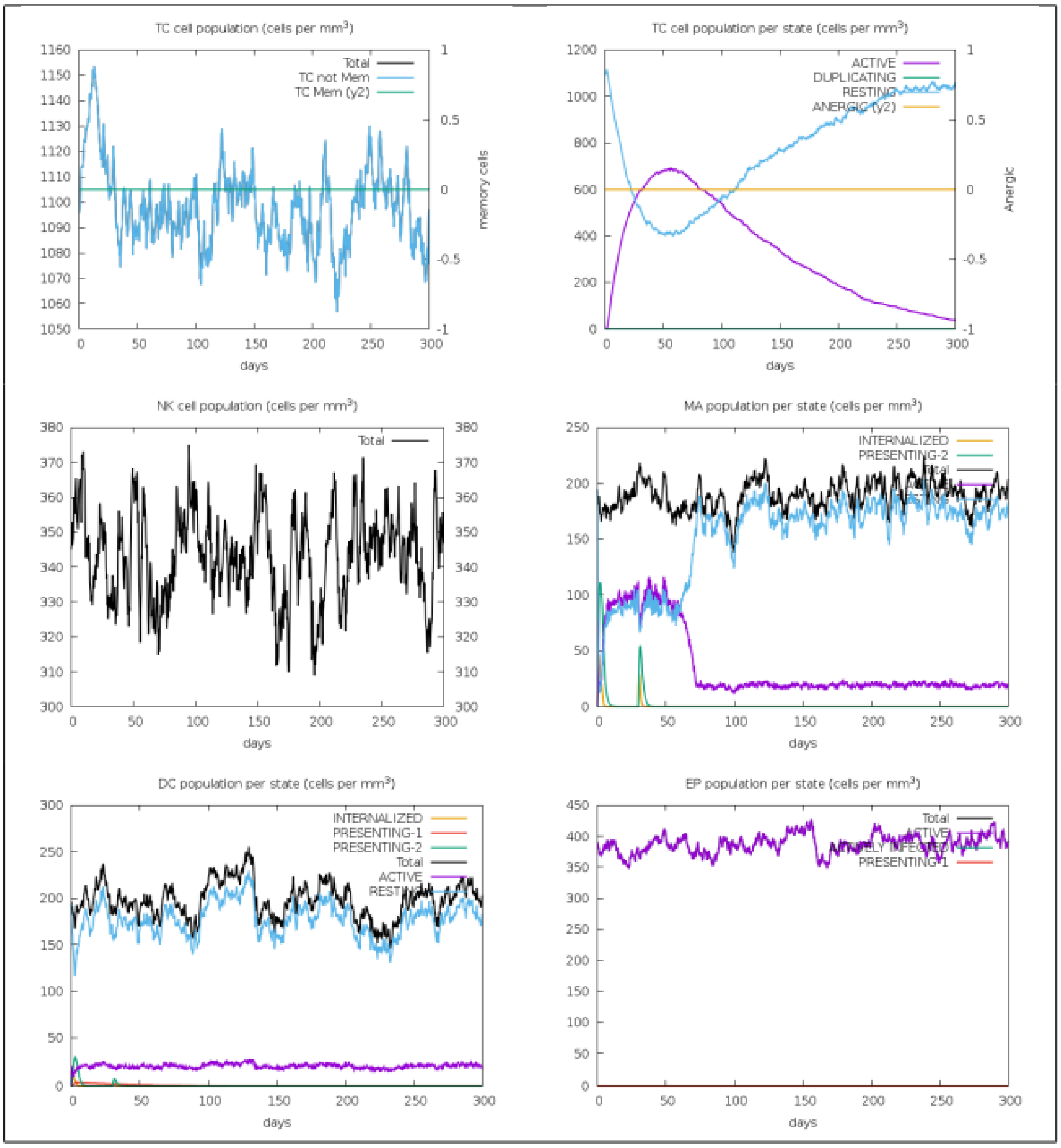

Effect of proposed vaccine on cell population

Figure S1. Results of Immune simulation after the first booster dose.

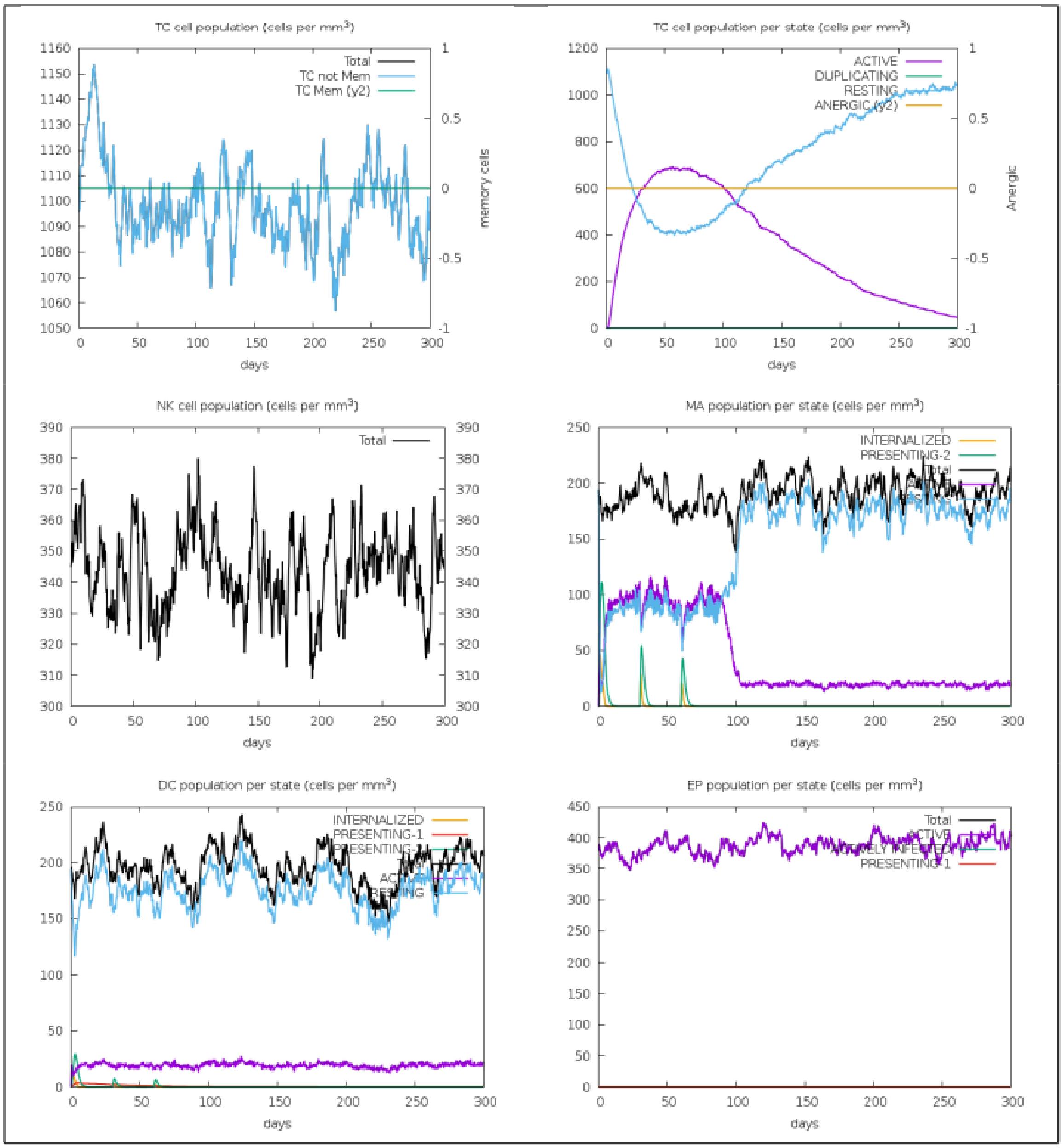

Effect of final dose of proposed vaccine on T-cell population

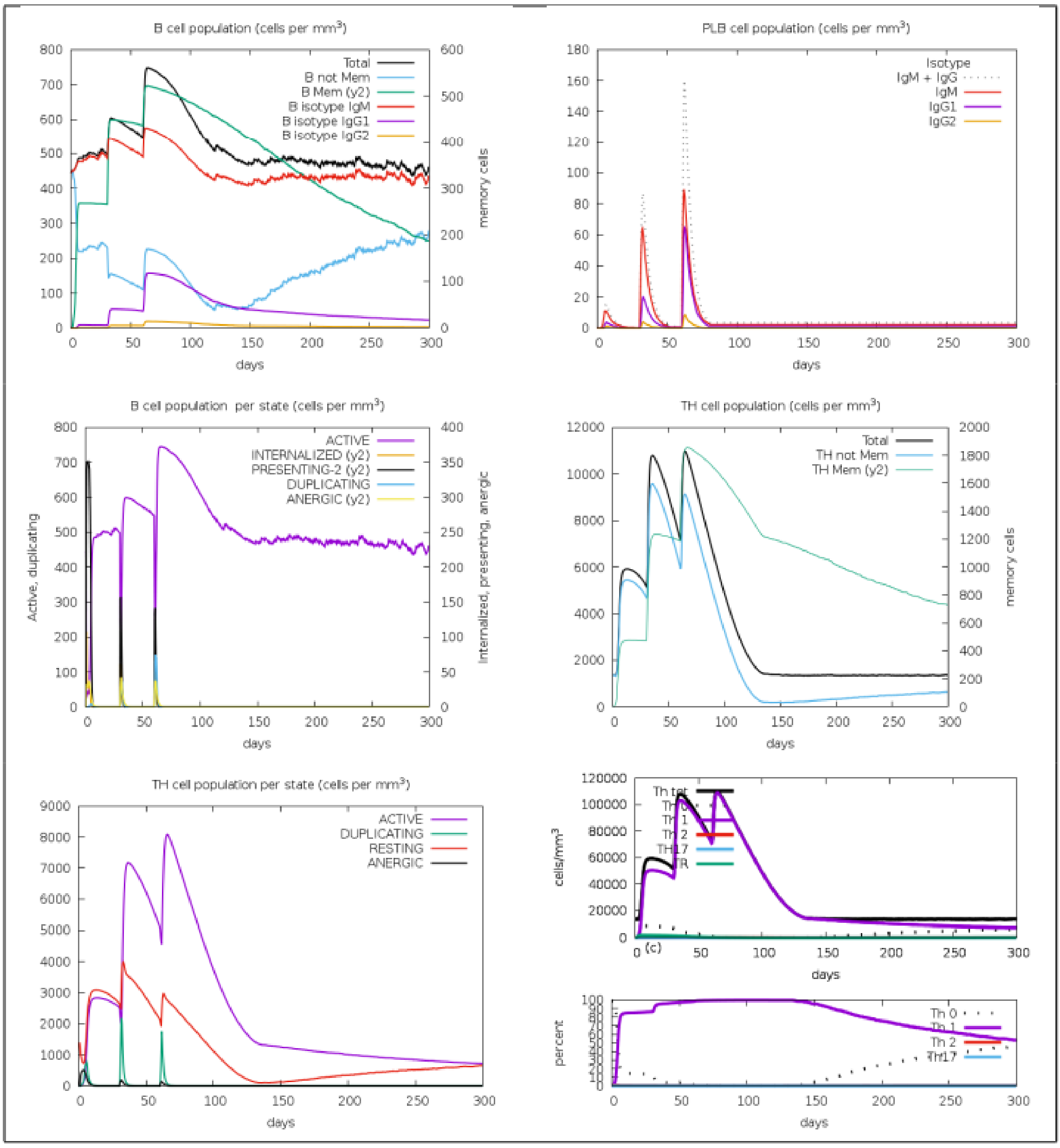

Effect of final dose of proposed vaccine on B-cell population

Figure S2. Effect of proposed vaccine on populations of immune cells after final booster dose.
