# Supplementary material for: Design and Validation of a Multi-Epitope mRNA Vaccine Construct Against Human Monkeypox Virus (hMPXV) by Annotating Protein of Intracellular Mature Virus (IMV) Form of hMPXV
Source: Biomedicines. 2025 Jun 11;13(6):1439. doi: 10.3390/biomedicines13061439 (PMC12190101; doi:10.3390/biomedicines13061439)
Supplement: Supplementary file 1 [file biomedicines-13-01439-s001.zip › Supplementary File S3.pdf]

JCat

JAVA Codon Adaptation Tool

Bioinformatic tools from our team:

PRODORIC Release 2

JCat was published in NAR (Nucleic Acids Research).

CodonUsage adapted to Escherichia coli (strain K12)

Improved DNA:

```
GAAGCTGCTGCTAAAGGTATCATCAACACCCTGCAGAAATACTACTGCCG      50
TGTTCGTGGTGGTCGTTGCGCTGTTCTGTCTTGCCTGCCGAAAGAAGAAC      100
AGATCGGTAAATGCTCTACCCGTGGTCGTAAATGCTGCCGTCGTAAAAAA      150
GAAGCTGCTGCTAAAGCTAAATTCGTTGCTGCTTGGACCCTGAAAGCTGC      200
TGCTGGTGGTGGTTCTACCCTGAAACAGCGTCTGACCAACCTGGAAAAAA      250
AAATCACCAACATCGGTCCGGGTCCGGGTAAAAAAAACCTGGAAAAAAAA      300
ATCACCAACATCACCACCAAATTCGAACAGGCTGCTTACACCCTGAAACA      350
GCGTCTGACCAACCTGGGTCCGGGTCCGGGTAAAAAAAAACAGCGTCTGA      400
CCAACCTGGAAAAAAAAATCACCAACATCACCGGTGGTGGTTCTCACGAA      450
TACGGTGCTGAAGCTCTGGAACGTGCTGGTGGTGGTGGTTCTCTGGTTCG      500
TATCAACTTCAAAGGTGGTTACATCTCTGGTGGTTTCCTGGGTCCGGGTC      550
CGGGTAAAAAAACCCTGGACATCCACTACAACGAATCTAAACCGACCACC      600
ATCCAGGCTGCTTACTCTGACCTGCGTGAAGCTTGCTTCTCTTACGGTCC      650
GGGTCCGGGTAAAAAACTGGAAAAAAAAATCACCAACATCACCACCAAAT      700
TCGAACAGATCGGTGGTGGTTCTCACGAATACGGTGCTGAAGCTCTGGAA      750
CGTGCTGGTGGTGGTGGTTCTCCGAACTTCTGGTCTCGTATCGGTACCGT      800
TGCTGCTAAACGTTACCCGGGTCCGGGTCCGGGTAAAAAACCGGACCACT      850
ACAAAGACTACGTTTTCATCCAGTGGACCGGTGGTGCTGCTTACCGTATC      900
GGTACCGTTGCTGCTAAACGTTACGGTGGTGGTTCTCACGAATACGGTGC      950
TGAAGCTCTGGAACGTGCTGGTGGTGGTGGTTCTGACGAATGCTACGGTG      1000
CTCCGGGTTCTCCGACCAACCTGGAATTCATCGGTCCGGGTCCGGGTAAA      1050
AAAAACGACAAAATCAAACTGATCCTGGCTAACAAAGAAAACGTTCACGC      1100
TGCTTACCTGACCCCGGAACAGAAAGCTTACGTTCACGAATACGGTGCTG      1150
AAGCTCTGGAACGTGCTGGTGCTAAATTCGTTGCTGCTTGGACCCTGAAA      1200
GCTGCTGCTGGTGGTGGTTCT
```

CAI-Value of the improved sequence:

1.0

GC-Content of the improved sequence:

50.859950859950864

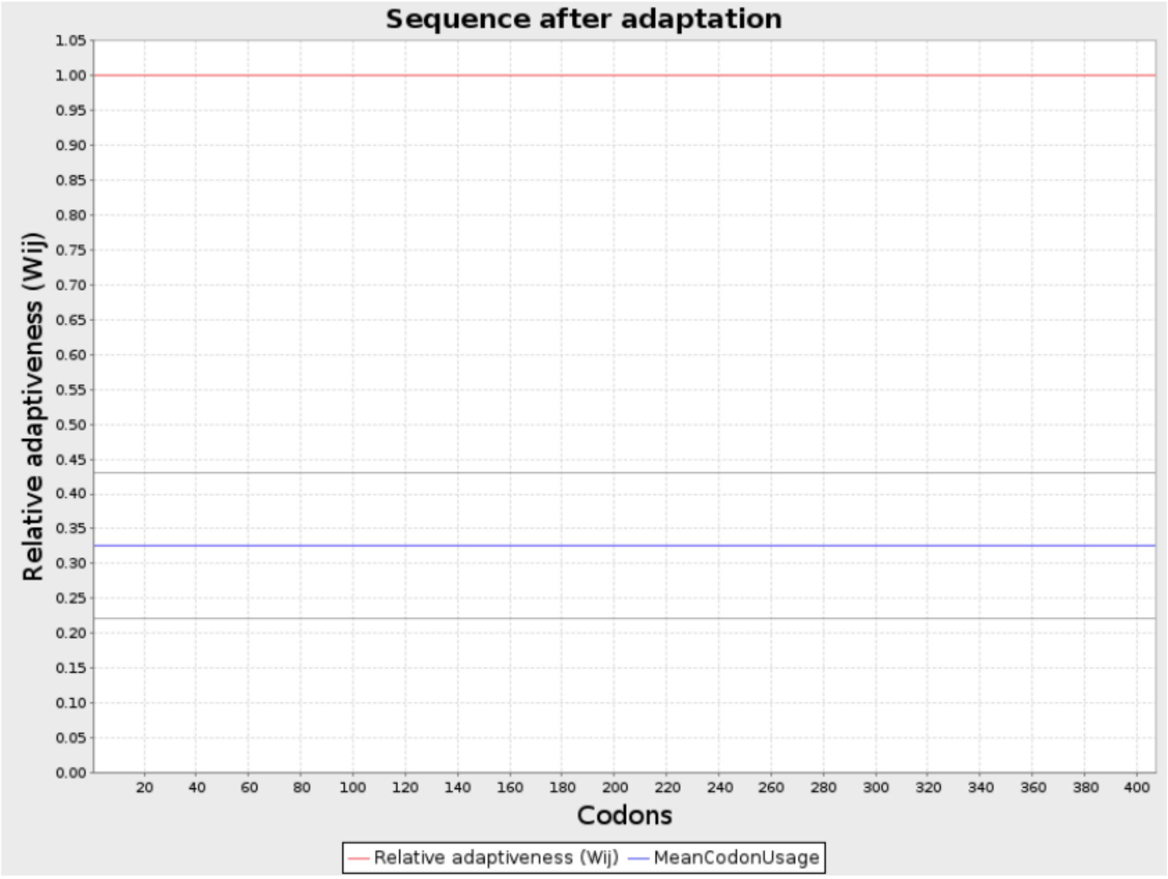

Figure S1. codon adaptation results.

Translation:

```
EAAAKGIINTLQKYYCRVRGGRCAVLSCLPKEEQIGKCSTRGRKCCRRKK     50
EAAAKAKFVAAWTLKAAAGGGSTLKQRLTNLEKKITNIGPGPGKKNLEKK    100
ITNITTKFEQAAYTLKQRLTNLGPGPGKKKQRLTNLEKKITNITGGGSHE    150
YGAEALERAGGGGSLVRINFKGGYISGGFLGPGPGKKTLDIHYNESKPTT    200
IQAAYSDLREACFSYGPGPGKKLEKKITNITTKFEQIGGGSHEYGAEALE    250
RAGGGGSPNFWSRIGTVAAKRYPGPGPGKKPDHYKDYVFIQWTGGAAYRI    300
GTVAAKRYGGGSHEYGAEALERAGGGGSDECYGAPGSPTNLEFIGPGPGK    350
KNDKIKLILANKENVHAAYLTPEQKAYVHEYGAEALERAGAKFVAAWTLK    400
AAAGGGS
```

EAAAKGIINTLQKYYCRVRGGRCAVLSCLPKEEQIGKCSTRGRKCCRRKKEAAAKA
KFVAAWTLKAAAGGGSTLKQRLTNLEKKITNIGPGPGKKNLEKKITNITTKFEQAAY
TLKQRLTNLGPGPGKKKQRLTNLEKKITNITGGGSHEYGAEALERAGGGGSLVRINF
KGGYISGGFLGPGPGKKTLDIHYNESKPTTIQAAYSDLREACFSYGPGPGKKLEKKIT
NITTKFEQIGGGSHEYGAEALERAGGGGSPNFWSRIGTVAAKRYPGPGPGKKPDHYK
DYVFIQWTGGAAYRIGTVAAKRYGGGSHEYGAEALERAGGGGSDECYGAPGSPTN
LEFIGPGPGKKNDKIKLILANKENVHAAYLTPEQKAYVHEYGAEALERAGAKFVAA
WTLKAAAGGGS
